# TXSelect: A multi-task learning model to identify secretory effectors

**DOI:** 10.1371/journal.pcbi.1013677

**Published:** 2025-11-06

**Authors:** Jing Li, Qing Liu, Quan Zou, Chao Zhan

**Affiliations:** 1 Department of Microbiology, University of Hong Kong, Hong Kong, China; 2 Yangtze Delta Region Institute (Quzhou), University of Electronic Science and Technology of China, Quzhou, China; 3 School of Biomedical Sciences, University of Hong Kong, Hong Kong, China; 4 Department of Anesthesiology, Hospital (T.C.M) Affiliated To Southwest Medical University, Luzhou, China; 5 Department of Hepatopancreatobiliary Surgery, Harbin Medical University Cancer Hospital, Harbin, China; Tel Aviv University, ISRAEL

## Abstract

Secretory effectors from pathogenic microorganisms significantly influence pathogen survival and pathogenicity by manipulating host signalling, immune responses, and metabolic processes. However, because of sequence and structural heterogeneity among bacterial effectors, accurately classifying multiple types simultaneously remains challenging. Therefore, we developed TXSelect, a multi-task learning framework that simultaneously classifies TXSE (types I, II, III, IV and VI secretory effectors) using a shared backbone network with task-specific heads. TXSelect integrates the protein embedding features of evolutionary scale modelling (ESM), particularly the N-terminal mean, with classical descriptors to effectively capture complementary information. These descriptors include distance-based residue (DR) and split amino acid composition general (SC-PseAAC-General). Rigorous evaluation identified ESM N-terminal mean + DR + SC-PseAAC as the optimal feature combination, achieving high accuracy (validation F1 = 0.867, test F1 = 0.8645) and robust generalization. Comprehensive assessments and visualization with Uniform Manifold Approximation and Projection further validated the discriminative capability and interpretability of the model. TXSelect provides an efficient computational tool for accurately classifying bacterial effectors, supporting deeper biological understanding and potential therapeutic development.

## Author summary

Secretory effectors are specialized proteins produced by pathogenic bacteria that allow them to infect host organisms by disrupting normal cellular functions. Accurately identifying and classifying these effectors is crucial for understanding infection mechanisms and developing new treatments, but this task is complicated by the high diversity in their sequences and structures. Here, we present TXSelect, a new artificial intelligence model that uses multi-task deep learning to simultaneously recognize multiple types of bacterial secretory effectors. Our model combines advanced protein sequence embeddings from large-scale evolutionary models with classical biochemical descriptors, enabling it to capture more information than either method alone. We rigorously evaluated TXSelect using multiple datasets and strict experimental protocols, demonstrating that it achieves high accuracy and robust performance even under challenging scenarios where the similarities between training and test samples are minimized. Additionally, our analyses provide interpretable insights into which molecular features are most important for distinguishing different effector types.

## 1. Introduction

Secretory Effectors are protein molecules secreted by various pathogenic microorganisms during host infection [1,2]. They significantly influence pathogen survival and proliferation by manipulating host signalling pathways, immune responses, and metabolic processes [3]. Depending on the secretion systems utilized by bacteria, effectors are classified into Type I–VII secretory effectors [4], each with distinct roles in host-pathogen interactions. Type I secretory effectors (T1SE) are typically secreted directly across the bacterial membrane into the extracellular environment via ABC transporter protein complexes. In this environment, they mediate toxicity, antimicrobial activity, or immune evasion [5]. These effectors typically possess simple and clear sequence signals. Type II secretory effectors (T2SE) rely on the complex Type II secretion system for extracellular transport, participating in degrading host cell wall components or modulating host defences [6]. Type III secretory effectors (T3SE) are directly injected into host cells by a specialized needle-like injectisome. They broadly disrupt host immune signalling pathways and cellular functions, and are critical pathogenic factors during bacterial infection [7]. Type IV secretory effectors (T4SE) are transferred into host cells or other bacteria through the Type IV secretion system. They mediate horizontal transfer of DNA, proteins, and metabolites, thereby influencing host cell signalling and immune regulation [8]. Type V secretory effectors (T5SE) use an autotransporter mechanism, where the proteins themselves traverse the bacterial cell membrane independently of other transporter proteins [9]. These autotransporter proteins commonly facilitate host adhesion and invasion, significantly contributing to pathogenesis. Type VI secretory effectors (T6SE) are delivered through the needle-tube-like Type VI secretion system, used either for competing with commensal bacteria or directly attacking host cells [10]. Type VII secretory effectors (T7SE) utilize a specialized Type VII secretion system. This system has been extensively studied in *Mycobacterium* species and mediates pathogen-host immune system interactions, contributing significantly to immune evasion and infection processes [11].

Although T5SE and T7SE hold important theoretical value in understanding pathogenic mechanisms, we were unable to obtain sufficiently high-quality, low-redundancy, and clearly annotated sequences for these effectors. This was primarily due to data complexity, sample source diversity, unclear annotations, and high redundancy in public databases and literature-reported sequences [12–14]. Therefore, in this study, we focused on T1/2/3/4/6SE, which have more reliable data quality and clearer sequence characteristics. These effectors have been extensively studied, ensuring accuracy and reliability in model training. We collectively refer to T1/2/3/4/6SE as TXSE. Studying the biological characteristics and pathogenic mechanisms of different types of effectors is critical for gaining deeper insights into bacterial pathogenesis and for developing antibacterial drugs or therapeutic strategies [15]. However, significant heterogeneity exists across effector types in terms of sequence composition, structural features, and functional mechanisms, presenting considerable challenges for computational methods.

Several computational tools and models have been developed for identifying specific effector types, such as Bastion3 [16] and DeepT3 [17] for T3SE, and T4SEfinder [18] and DeepSecE [19] for T4SE, with some success in single-task identification. Recent methods such as TSE-ARF [20] and PREFFECTOR [21] have extended their prediction capability across multiple effector types or secretion systems. However, these methods are fundamentally multi-class approaches rather than explicitly structured multi-task learning frameworks. Consequently, they cannot simultaneously capture task-specific characteristics and shared representations. Furthermore, the high sequence heterogeneity across effector types limits the efficiency and accuracy of single-task and multi-class models in concurrent classification. Additionally, existing models lack strategies to comprehensively utilize diverse sequence features and exploit complementary information from various feature representations, restricting their overall generalization performance. To address these challenges, explicitly designed computational methods for accurate and simultaneous identification of multiple effector types remain crucial. Multi-task learning, a machine learning paradigm capable of capturing both commonalities and differences between tasks, has achieved considerable success in bioinformatics. However, this approach remains underexplored in bacterial secretory effector classification.

In this study, we propose a multi-task learning model named TXSelect, designed to efficiently and accurately identify different effector types (T1/2/3/4/6SE) by integrating a shared feature extraction network with task-specific classification heads. The TXSelect model systematically integrates protein embedding features from evolutionary scale modelling (ESM) with distance-based residue (DR) and split amino acid composition (SC-PseAAC), leveraging their complementary advantages. Specifically, the innovations and contributions of this study include: (1) construction of a multi-task learning framework capable of simultaneously classifying multiple bacterial effector types, effectively addressing the limited generalization performance of traditional single-task classification models; (2) development of a comprehensive feature evaluation and fusion strategy, which systematically analyses and identifies the optimal combination of ESM features (particularly the N-terminal region) and classical sequence descriptors, significantly enhancing prediction accuracy; and (3) implementation of extensive hyperparameter tuning and structural optimization to establish an efficient and robust TXSelect model, providing essential theoretical methodologies and practical references for future studies on bacterial effector identification.

## 2. Results

### 2.1. Feature importance and selection

#### 2.1.1. Evaluation of ESM pooling strategies.

**Fig 1:**
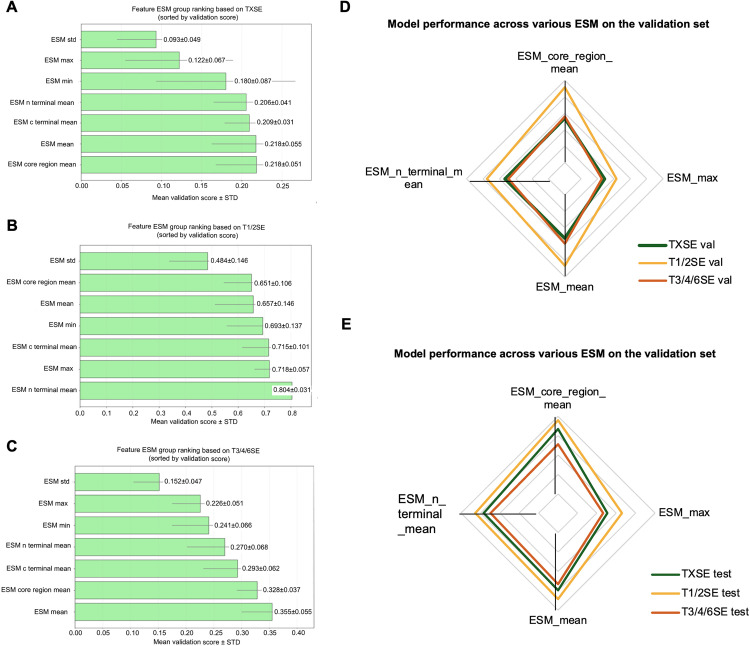
ESM group ranking and performance of selected ESM pooling strategies in multi-task classification. (A–C) Feature group ranking based on silhouette scores. Supervised Uniform Manifold Approximation and Projection (UMAP) with 5-fold cross-validation was used to evaluate clustering ability of different ESM pooling strategies across tasks. Bars indicate the mean validation silhouette score ± standard deviation for (A) TXSE (T1/2/3/4/6SE), (B) T1/2SE subset, and (C) T3/4/6SE subset. (D–E) Performance of selected ESM features. Based on ranking results and widely recognized pooling strategies, ESM mean, ESM max, ESM N-terminal mean, and ESM core region mean were selected for multi-task training. Radar plots show their classification performance across TXSE, T1/2SE, and T3/4/6SE tasks on the (D) validation set and (E) test set. Among these, ESM core region mean, ESM N-terminal mean, and ESM mean consistently achieved strong performance.

To identify the most suitable ESM features for the TXSE multi-task classification, we ranked and evaluated various ESM pooling strategies based on the Silhouette Score [22] obtained after dimensionality reduction using Uniform Manifold Approximation and Projection [22,23]. Fig 1A–C show the comparative performance of different ESM features for the TXSE task as well as for the T1/2SE and T3/4/6SE sub-tasks. Overall, the different ESM pooling strategies showed significant variations across different task scenarios.

**TXSE task:** As shown in Fig 1A, the ESM core region mean and ESM mean performed best (validation scores = 0.218 for both), followed by ESM C-terminal mean (0.209) and ESM N-terminal mean (0.206). In comparison, ESM min, ESM max, and ESM std performed poorly, with ESM std (0.093) showing the lowest performance.

**T1/2SE sub-task:** As shown in Fig 1B, the discriminatory capability of all pooling methods was generally higher than that of the overall TXSE task. Among these, ESM N-terminal mean achieved the best performance (0.804), followed by ESM max (0.718) and ESM C-terminal mean (0.715), while ESM std exhibited the lowest performance (0.484).

**T3/4/6SE sub-task:** As shown in Fig 1C, ESM mean performed best (0.355), followed by ESM core region mean (0.328). While ESM C-terminal mean (0.293) and ESM N-terminal mean (0.270) showed intermediate results, ESM std (0.152) was again the weakest.

From these analyses, we found that region-specific pooling methods (such as ESM n-terminal mean and ESM core region mean) generally performed better, especially in sub-tasks. In contrast, ESM std consistently showed the poorest performance across all tasks, indicating that using standard deviation alone contributes limited discriminatory power for effector identification. Furthermore, the overall discrimination effectiveness of the T1/2SE task was clearly higher than that of the T3/4/6SE task, suggesting more complex feature differences in T3/4/6SE, which makes clustering and discrimination more challenging. In conclusion, region-specific features (ESM N-terminal mean, ESM core region mean) and global average features (ESM mean) should be prioritized for subsequent feature fusion in multi-task modelling, as they enhance both prediction stability and generalization.

#### 2.1.2. Multitask model performance based on selected ESM features.

To validate the practical effectiveness of the different ESM pooling strategies in multi-task classification, we selected the ESM N-terminal mean, ESM core region mean and ESM mean—the top-performing strategies from the feature ranking—for model construction and evaluation. Additionally, since ESM max has demonstrated strong generalization performance in other research contexts, we also included it in comparative evaluations to achieve a more comprehensive performance analysis [24].

Fig 1D and 1E show radar plots representing the classification performance (F1 scores) of multi-task models based on different ESM feature strategies across validation and test sets, respectively. On the validation set (Fig 1D), for the TXSE task, the performances of the four features were comparable, although ESM N-terminal mean and ESM mean performed slightly better. For the T1/2SE sub-task, ESM N-terminal mean achieved the best performance, clearly surpassing the other features. For the T3/4/6SE sub-task, ESM mean and ESM core region mean performed best, while ESM max was slightly inferior. On the test set (Fig 1E), ESM N-terminal mean significantly outperformed other feature strategies in both the TXSE task and T1/2SE sub-task, demonstrating strong generalization capabilities. For the T3/4/6SE sub-task, both ESM mean and ESM core region mean maintained high performance, reinforcing their applicability in complex task scenarios.

In summary, ESM N-terminal mean, ESM core region mean, and ESM mean consistently outperformed ESM max across both validation and test sets, highlighting their potential for further research. Subsequent model construction will therefore focus on these features to achieve more efficient and accurate classification predictions.

#### 2.1.3. Evaluation of classical sequence descriptors (TXSE, T1/2SE, T3/4/6SE).

**Fig 2:**
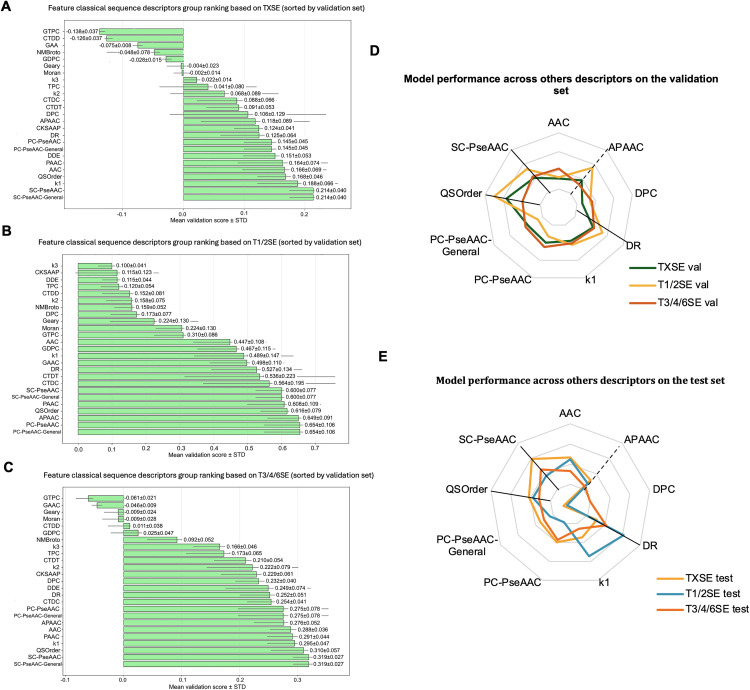
Classical sequence descriptor group ranking and performance of selected descriptors in multi-task classification. (A–C) Feature group ranking based on silhouette scores. Supervised UMAP with 5-fold cross-validation was applied to evaluate the clustering ability of various handcrafted sequence descriptors. Bars indicate the mean validation silhouette score ± standard deviation for (A) TXSE (T1/2/3/4/6SE), (B) T1/2SE subset, and (C) T3/4/6SE subset. (D–E) Performance of selected descriptors. Radar plots summarize the classification performance of representative descriptors (DR, SC-PseAAC, PC-PseAAC, QSOrder, AAC, and APAAC) across tasks. Results are shown for the (D) validation set and (E) test set. Among these, DR, SC-PseAAC, and QSOrder consistently achieved strong performance across tasks.

To comprehensively explore the contributions of different feature types to effector classification tasks, we evaluated the performance of several classical sequence descriptors [25,26] across the TXSE task and T1/2SE and T3/4/6SE sub-tasks. Feature ranking was performed based on cross-validation results (Fig 2A–C).

**TXSE task:** As shown in Fig 2A, the best-performing descriptors mainly included SC-PseAAC-General, SC-PseAAC [25,26], k1 [27], and QSOrder [28]. Among them, SC-PseAAC-General (0.214 ± 0.040) and SC-PseAAC (0.214 ± 0.040) demonstrated identical and superior performance on the validation set.

**T1/2SE sub-task:** As shown in Fig 2B, the top four descriptors were PC-PseAAC-General (0.654 ± 0.106), PC-PseAAC (0.654 ± 0.106), APAAC (0.649 ± 0.091), and QSOrder (0.616 ± 0.079). These descriptors exhibited good generalization performance, with PC-PseAAC-General and PC-PseAAC significantly outperforming classical sequence descriptors on the validation set.

**T3/4/6SE sub-task:** As shown in Fig 2C, the prominent descriptors included SC-PseAAC-General (0.319 ± 0.027), SC-PseAAC (0.319 ± 0.027), QSOrder (0.310 ± 0.057), and k1 (0.295 ± 0.047), with SC-PseAAC-General and SC-PseAAC showing equally superior performance.

#### 2.1.4. Multitask model performance based on classical descriptors.

Making a comprehensive assessment of the feature ranking results from the TXSE task, T1/2SE and T3/4/6SE sub-tasks, we selected SC-PseAAC, QSOrder, PC-PseAAC-General, PC-PseAAC, k1, and APAAC, which ranked highly and demonstrated stable cross-task performance, to conduct further multi-task classification modelling research. Our previous T4Seeker study [29] highlighted the outstanding performance of the DR descriptor in T4SE identification. Moreover, AAC and DPC features performed well in the multi-classification task for T3/4/6SE. Therefore, we included these features in the current multi-task model evaluation.

Figs 2D and 4E present the F1 score performance of various classical descriptor for multi-task models on the validation and test sets, respectively. The individual of QSOrder, SC-PseAAC, and DR achieved the best overall performance on both sets. Notably, although APAAC performed well on the validation set, its performance significantly declined on the test set, indicating relatively weaker generalization capability. Therefore, it was excluded from subsequent feature fusion research stages. In summary, QSOrder, SC-PseAAC, and DR were selected for the next stage of analysis to optimize the model and conduct feature fusion research.

**Fig 3:**
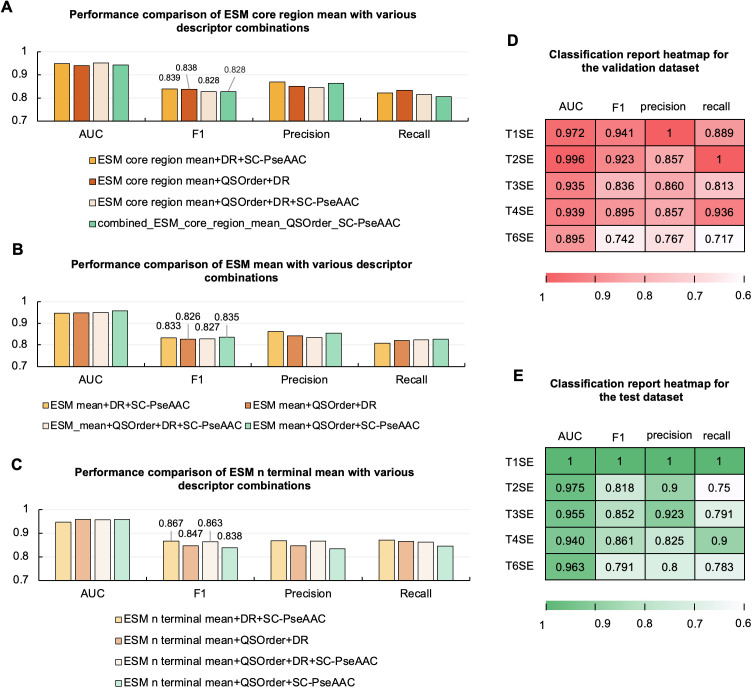
Performance comparison of feature combinations and detailed performance of the optimal TXSelect framework. (A–C) Model performance based on combinations of selected ESM pooling and classical sequence descriptors. Fusion experiments were conducted by combining ESM core region mean, ESM mean, and ESM N-terminal mean with DR, SC-PseAAC, and QSOrder. Bars indicate classification metrics (AUC, F1, Precision, Recall). Among these, ESM N-terminal mean + DR + SC-PseAAC achieved the highest validation F1 score (0.867) and also performed best on the test set (F1 = 0.8645). Adding QSOrder resulted in a comparable validation F1 score (0.863); however, its test performance decreased (F1 = 0.8507), confirming ESM N-terminal mean + DR + SC-PseAAC as the optimal combination. (D–E) Classification performance of the optimal TXSelect. Heatmaps show per-class performance of the optimal feature combination (ESM N-terminal mean + DR + SC-PseAAC) on the (D) validation dataset and (E) test dataset. Metrics (AUC, F1, Precision, Recall) are reported for each effector type (T1SE, T2SE, T3SE, T4SE, T6SE).

### 2.2. Feature combination improves multitask performance

To further improve the multi-task classification model, we evaluated its performance after combining ESM features (ESM core region mean, ESM mean, ESM N-terminal mean) with classical sequence descriptors (DR, SC-PseAAC, QSOrder) (Fig 3A–C) [30,31].

For ESM core region mean (Fig 3A), combining it with classical descriptors yielded overall F1 scores ranging 0.828–0.839, exhibiting stable performance (although not significantly improved). Similarly, for ESM mean (Fig 3B), the fusion resulted in performance fluctuations, with F1 scores ranging 0.826–0.835. Overall, both combinations showed limited performance improvement and minor differences between combined features. In comparison, combining ESM N-terminal mean with classical descriptors (Fig 3C) demonstrated a clear performance advantage. Specifically, ESM N-terminal mean + DR + SC-PseAAC achieved the highest F1 score (0.867) on the validation set and showed the best performance on the test set (F1 = 0.8645), demonstrating its excellent generalization capability and robustness. Notably, adding the QSOrder descriptor to this combination (ESM N-terminal mean + DR + SC-PseAAC + QSOrder) yielded an F1 score of 0.863 on the validation set, close to the optimal combination. However, its performance decreased on the test set (F1 = 0.8507), indicating that introducing QSOrder may lead to overfitting without improving generalization [32,33].

In summary, ESM N-terminal mean + DR + SC-PseAAC consistently delivered the best performance across validation and test sets, and was identified as the optimal feature combination with the best generalization for subsequent modelling and analysis. The detailed scatter plots for ESM N-terminal mean, DR, and SC-PseAAC are shown in S1 Fig.

### 2.3. Final multitask model evaluation

#### 2.3.1. Comprehensive performance evaluation of the final multitask model.

The optimal feature combination identified in this study was ESM N-terminal mean + DR + SC-PseAAC, demonstrating overall superior performance on both the validation and test sets. Fig 3D and 3E show heatmaps presenting the classification performance of this optimal model on the validation set and test set, respectively, evaluated using AUC, F1-score, Precision, and Recall.

On the validation set (Fig 3D), the model performed exceptionally well for T1SE and T2SE. Specifically, it showed extremely high precision for T1SE (precision = 1) and an F1 score of 0.941, indicating accurate and stable identification. For T2SE, it exhibited excellent recall (recall = 1), demonstrating effective capture of all samples in this category. The classification performances for T3SE and T4SE were robust and similar, with F1 scores of 0.836 and 0.895m and AUC values of 0.935 and 0.939, respectively, indicating stable and reliable classification capability for these two effector types. Comparatively, T6SE identification was more challenging, with a relatively lower F1 score (0.742). This suggests that the sequence characteristics of T6SE might be less distinct, hindering accurate differentiation. On the test set (Fig 3E), the model achieved perfect classification performance for T1SE (AUC = 1, precision = 1, recall = 1), further confirming its excellent generalization capability for classes with clearly defined sequence features [34]. However, for T2SE, the F1 score decreased slightly on the test set (0.818), mainly due to reduced recall (0.75). This indicates that the model experienced some missed detections. Nevertheless, the performance for T3SE and T4SE remained robust, with F1 scores of 0.852 and 0.861, respectively. For T6SE, the F1 score (0.791) improved slightly with the test set compared to that with the validation set, reflecting the resilience and generalization capabilities of the model.

In summary, considering the overall performance on both validation and test sets, the multi-task model constructed using the ESM N-terminal mean + DR + SC-PseAAC combination exhibited strong accuracy, sensitivity, and generalization abilities. Particularly, it excelled in identifying T1SE and T2SE with clearly defined sequence characteristics, thus providing a solid reference for improving identification methods for more challenging effector categories.

#### 2.3.2. Training stability and convergence analysis.

**Fig 4:**
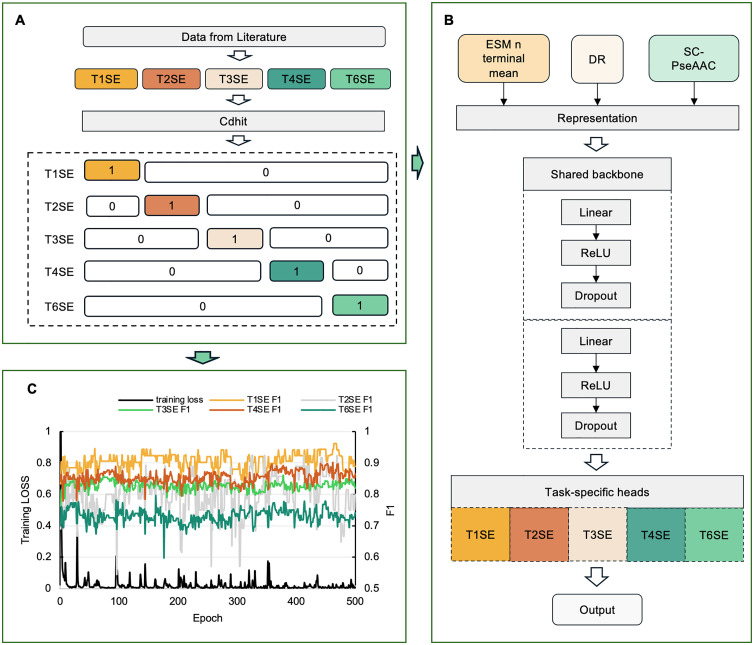
Overview of the TXSelect framework for multi-task identification of secretory effectors. (A) Dataset construction. Secretory effectors (T1SE, T2SE, T3SE, T4SE, and T6SE) were collected from the literature and redundancy was removed using CD-HIT. For each task, the target label was set to 1, whereas the labels of the other tasks were set to 0 (e.g., in the T1SE task, the T1SE label is 1, while the labels for T2/3/4/6SE are 0). (B) Model architecture. Multiple feature descriptors, including evolutionary scale modelling. (ESM) N-terminal mean embedding, distance-based residue (DR), and split amino acid composition (SC-PseAAC), were integrated to construct sequence representations. These representations are processed through a shared backbone network composed of stacked linear, ReLU, and dropout layers, followed by task-specific heads for predicting different effector types. (C) Model performance. Training loss and validation F1-scores of T1SE, T2SE, T3SE, T4SE, and T6SE tasks across 500 epochs. The curves demonstrate stable convergence of the shared multi-task framework and balanced performance across effector classes.

The flowchart of the model is shown in Fig 4, with data processing presented in Fig 4A and model construction shown in Fig 4B. The training process of the multi-task classification model is illustrated in Fig 4C. The training utilized cross-entropy loss and the Adam optimizer, running for a total of 500 epochs [35,36]. During each epoch, we recorded the training loss on the training set and the F1 score trends for each task (T1SE, T2SE, T3SE, T4SE, and T6SE) on the validation set.

As shown in the Fig 4C, the training loss rapidly decreased within the first 50 epochs and then stabilized at a low level, indicating that the model converged quickly and exhibited stability [37]. Regarding classification performance on the validation set, each task demonstrated varying degrees of stability and performance. The F1 scores for tasks T1SE and T2SE were relatively high, remaining consistently above 0.85. The T1SE task particularly showed the most stable performance. Tasks T4SE and T3SE exhibited relatively stable performances, with their F1 scores maintained around 0.85 and 0.80, respectively. This indicates that the model consistently recognized these two effector types. However, the T6SE task demonstrated relatively lower and more fluctuating F1 scores, stabilizing around 0.75, suggesting challenges due to elusive sequence features [38]. Details of the hyperparameter tuning process and the impact on model performance are shown in S2 Fig. Feature attribution heatmaps can be found in S3–S7 Figs, highlighting the contribution of individual features. The top 20 most important features ranked by SHAP values are depicted in S8–S12 Figs. We have conducted additional experiments under a homology-controlled evaluation setting, using a 50% CD-HIT cut-off for splitting the train, validation, and test sets. The data distribution is summarized in Table 1. The model training curves are illustrated in S13 Fig, and the corresponding results are presented in S14 Fig.

**Table 1:** Data sources and sample sizes for each effector category.

Data type	Data source	Sample number after CD-HIT	
		Cut-off = 80%	cut-off = 50%
T1SE	TSE-ARF [20]	175	135
T2SE	TSE-ARF [20]	80	76
T3SE	DeepT3 [40] Bastion3 [16] TSE-ARF [20]	606	496
T4SE	DeepT3_4 [43] OPT4e [45] Bastion4 [47] DeepSecE [19] T4Sefinder [18] T4SE-XGB [48] T4SEpp [49] TSE-ARF [20] iTSE-EP [46]	730	626
T6SE	DeepSecET6 [41] TSE-ARF [20] Bastion6 [42]	308	250

Overall, the stable training loss combined with the consistent validation performance curves for each task demonstrates a reliable and stable model training process. The sustained and stable F1 scores further confirm the effectiveness of the model.

### 2.4. Comparison of TXSelect with an existing state-of-the-art method

To further evaluate the performance of the TXSelect multi-task model, we conducted a comparative analysis with an existing method, SBSM-Pro [39]. However, SBSM-Pro could not be directly applied to the multi-task classification scenarios in this study. Therefore, we treated it as a multi-class prediction task and evaluated its performance under the same dataset partitioning and feature conditions to ensure a fair comparison.

Under identical conditions and using the same training, validation, and test sets, the SBSM-Pro multi-class model achieved macro-average F1 scores of 51.54 and 55.50% with the validation and test sets, respectively. In contrast, our proposed multi-task model achieved significantly higher average F1 scores of 86.73 and 86.45% with the validation and test sets, respectively, indicating substantial performance improvement. Notably, for T4SE and T6SE, the SBSM-Pro model showed significantly lower F1 scores (approximately 12 and 52%, respectively), whereas the proposed model achieved approximately 89.5 and 74.2%, respectively. This clearly demonstrates the considerable advantage of the multi-task learning framework in handling categories with sparse samples.

Moreover, we considered additional recent multi-effector prediction methods, including TSE-ARF [20] and PREFFECTOR [21], for comparative analysis. However, due to the unavailability of publicly accessible model code and detailed implementation descriptions, direct performance comparisons with these methods were not feasible.

## 3. Discussion

This study proposes TXSelect, a systematic, multi-task framework for predicting and classifying bacterial secretory effectors, which significantly enhances classification performance across different effector types. By designing a shared feature representation layer together with task-specific classification heads, this framework enables effective collaboration and information sharing among classification tasks, thereby improving the predictive performance and generalization capability of the model. In terms of feature design and combination, TXSelect systematically evaluated and validated the combined advantages of ESM-derived and classical sequence descriptors. We identified ESM N-terminal mean + DR + SC-PseAAC as the optimal feature combination strategy, which significantly improved the accuracy and robustness of effector classification. This multi-scale feature combination not only enhances model performance but also provides strong biological interpretability. Thus, it facilitates a deeper understanding of relationships between the effector sequence characteristics and functional mechanisms of bacteria.

While TXSelect demonstrates promising performance and generalization on the current benchmark datasets, there are several important limitations to note. First, as the framework is primarily based on sequence-derived representations, it inherently loses information related to the 3D stereochemistry and topological relationships of effector proteins, which may limit its ability to capture certain functional or structural features. This information loss may particularly impact the model’s performance on tasks requiring spatial or structural discrimination. In addition, the applicability of TXSelect to effectors from unknown bacterial species or clinical isolates requires further large-scale experimental verification. The current framework is most effective when high-quality sequence data are available, but the robustness in scenarios with low-quality or incomplete sequences remains to be established. Although strict data integration, de-redundancy, and uniform filtering criteria were applied to harmonize samples collected from multiple sources, the possibility of residual sampling bias or inconsistency cannot be entirely excluded. Although TXSelect shows promising results on current benchmark datasets, its performance on larger-scale datasets or previously unseen bacterial species remains to be systematically evaluated. As more data become available, future work will focus on: (1) Integrating additional structural and spatial features to compensate for the information loss from purely sequence-based representations; (2) incorporating additional types of bacterial secretory effectors (e.g., by obtaining high-quality cleaned data for T5/7SE); (3) exploring cross-species prediction to further enhance generalization performance; and (4) enhancing the interpretability of the model to reveal potential relationships between key effector sequences, structural features, and their biological functions. These research directions will further consolidate TXSelect as an effective tool for the computational prediction and bioinformatics analysis of bacterial secretory effectors.

## 4. Materials and methods

### 4.1. Data description

To effectively train a multi-task classification model capable of identifying different types of secretory effectors, we systematically integrated and constructed corresponding protein sequence datasets from multiple authoritative databases and published literature. For T1SE and T2SE, we used TSE-ARF data [20] to construct subsets. These sequences have undergone strict data validation and biological verification, making them suitable for building high-quality classification models. For T3SE and T6SE, we integrated multiple sources, including Bastion3 [16], DeepT3 [40], SecReT6 [41], Bastion6 [42], DeepT3_4 [43], and TSE-ARF [20]. After merging sequences across these databases, we initially obtained a rich set of candidate effector sequences. Redundancy was removed using the CD-HIT tool [44] with an 80% sequence similarity threshold, enhancing sequence representativeness and diversity. This ultimately yielded T3SE and T6SE sequence datasets suitable for model training. T4SE sequences were collected from multiple prediction platforms and databases, including DeepSecE [19], OPT4e [45], T4Sefinder [18], iT4SE-EP [46], Bastion4 [47], T4SE-XGB [48], TSE-ARF [20], T4Sepp [49], and DeepT3_4 [43]. Following data integration and standardization, CD-HIT (80% similarity threshold) was applied to eliminate high-sequence similarity, obtaining a concise and representative T4SE dataset. The details of data source, and sample sizes after CD-HIT for each effector category are summarized in Table 1.

After processing, we obtained a comprehensive multi-task dataset, with the specific data distribution for each effector type shown in Fig 4A. During data annotation, we employed the following labelling strategy to clearly illustrate the definitions in the multi-task learning framework. For each specific sub-task, samples of the current effector type were labelled as positive (label = 1), while the remaining four effector types were labelled as negative (label = 0). For example, during T1SE task training, T1SE samples were labelled as 1, whereas T2SE, T3SE, T4SE, and T6SE samples were uniformly labelled as 0. The same strategy was applied for T2SE, T3SE, T4SE, and T6SE, thus forming a multi-task classification system that can simultaneously train five sub-tasks (Fig 4A). To ensure balanced model training, we adopted a stratified sampling approach [50] according to the positive and negative sample distribution of different effector types. The data were partitioned into training, validation, and test sets at a ratio of 7:1.5:1.5, respectively, to be used for multi-task model training [51,52]. Through these procedures, we obtained a comprehensive, high-quality, low-redundancy dataset that effectively supports construction and optimization of the TXSelect multi-task classification model.

### 4.2. Feature extraction

#### 4.2.1. Global pooling.

**Fig 5:**
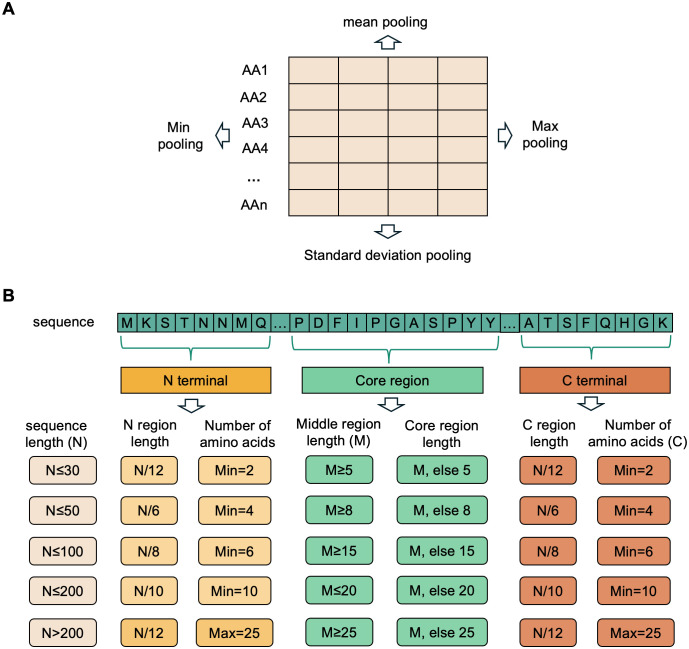
Feature process strategies for ESM representations. (A) Basic pooling operations, including mean, max, min, and standard deviation pooling, applied to residue-level embeddings. (B) Region-specific feature extraction. Protein sequences were divided into N-terminal, core region, and C-terminal segments according to sequence length. The lengths of terminal regions were dynamically determined with lower/upper constraints on amino acid counts. The remaining residues constituted the core region. Minimum length constraints were further applied to ensure balanced representation. Detailed rules for length assignment and minimum thresholds are provided in the Methods.

We also employed the advanced protein sequence embedding model, ESM [24,53], to extract feature representations from protein sequences. ESM is a pre-trained Transformer-based language model, which effectively captures structural, functional, and evolutionary features by learning from large-scale protein sequence datasets. Specifically, we used the ESM-2 model (650M parameters, as provided in the official Facebook AI ESM repository), which outputs 320-dimensional embedding vectors for each amino acid. For any given protein sequence of length N (number of amino acids), we processed the sequence using the pre-trained ESM model, which generates a set of high-dimensional vector representations for each amino acid position within the protein sequence. In this study, each amino acid position was represented as a feature vector of fixed dimension (320 dimensions), forming an embedding matrix of size (N × 320). Each row of this matrix corresponds to a specific amino acid position in the sequence, while each column represents a particular feature dimension extracted by the model. Since protein sequences vary in length, directly using the original ESM output matrices poses challenges for training the subsequent classification models. Therefore, we applied certain feature pooling strategies to transform these variable-length sequence representations into fixed-length, uniform-dimensional feature vectors, thus facilitating model training and feature integration. We adopted four widely used pooling methods (Fig 5A) to comprehensively capture potentially significant feature information within protein sequences [54]:

**Mean Pooling.** Computes the average value across each dimension of all amino acid embedding vectors, producing an overall average feature representation of the sequence. It smoothly captures the overall feature trends of sequences and is suitable for most feature representations (ESM mean).

**Max Pooling.** Takes the maximum value across all amino acid embeddings in each feature dimension, highlighting amino acid sites with strong signals or significant roles (ESM max).

**Min Pooling.** Selects the minimum value across all amino acid embeddings in each feature dimension, emphasizing features that are conserved or significantly lower, thereby reflecting an alternative dimension of the protein sequence characteristics (ESM min).

**Standard Deviation Pooling.** Calculates the standard deviation across all amino acid embeddings in each dimension, effectively representing the variability and heterogeneity in amino acid features (ESM std).

Together, these four basic pooling methods transform each protein sequence into four feature vectors of fixed dimensionality. These vectors comprehensively describe the global features of protein sequences from different statistical perspectives. The characterization provides a rich informational foundation for the multi-task classification model, enhancing its accuracy in identifying different types of effectors.

#### 4.2.2. Region-specific features.

Although global pooling effectively captures overall protein sequence features, previous studies have shown that different sequence regions possess distinct importance in structural formation and functional execution [55]. Therefore, we conducted a more refined analysis of region-specific features, specifically dividing sequences into three regions: N-terminal, C-terminal, and Core region. The detailed approach is as follows:

(1) Determination of N-terminal and C-terminal region lengths

We dynamically determined the number of amino acids in the N-terminal and C-terminal regions based on the total length of each protein sequence (N, total number of amino acids). This dynamic adjustment strategy ensures biologically meaningful feature representations regardless of sequence length variations. The specific methods are detailed in Fig 5B. Based on the sequence length rules, the lengths of the N-terminal and C-terminal regions were calculated separately, selecting the corresponding number of amino acids from the start and end of the sequence, respectively. If the computed length was lower than the minimum number of amino acids (Min), as specified in Fig 5B, the Min value was used. Conversely, if the sequence length exceeded 200 and the computed value surpassed the maximum number of amino acids (Max = 25), the Max value was used. For example, in a 48-residue sequence, the calculated N-terminal length is 48/6 = 8, which is greater than the minimum threshold of 4; thus, 8 amino acids are selected. In a 280-residue sequence, the calculated region length is approximately 280/12 ≈ 23, which is less than the maximum threshold of 25; thus, 23 amino acids are directly selected. However, if the sequence length is 400, the calculated length is 33, which exceeds the maximum threshold; therefore, 25 amino acids are selected.

(2) Determination of minimum core region length

To ensure sufficient amino acid length for effectively characterizing the core functional region of the protein, we defined a minimum length (Core min length) based on different sequence lengths. The specific settings are detailed in Fig 5B. For example, in a 70-residue sequence, the corresponding Core min length is 15 amino acids.

(3) Calculation of core region length

Core region length was calculated by first removing the previously determined N-terminal and C-terminal regions to obtain the length of the middle region in the sequence:


M=N − (N−terminal length + C−terminal length) (1)

Subsequently, the middle region length was compared with the previously defined Core min length, leading to two possible scenarios:

Case ①: If M ≥ Core min length, the middle region is directly designated as the core region.

Case ②: If M < Core min length, the core region is defined by a symmetrical expansion from the sequence midpoint towards both ends, until the required Core min length is achieved.

Using this method, we ensured that feature extraction for the core region consistently maintained a stable and biologically meaningful amino acid length, thus enhancing the model’s ability to capture features from the core functional region.

### 4.3. Model architecture

Fig 4 illustrates the overall framework of this study, where data processing and task definition methods are shown in Fig 4A, and the model architecture is presented in Fig 4B. The specific training procedure (Fig 4C) and its performance evaluation are detailed in the Results section. To address the complex and diverse sequence characteristics of TXSE, we constructed a multi-task learning model based on a shared feature extraction backbone with task-specific classification heads. First, we conducted an in-depth exploration of sequence characteristics for different effector protein types. Ultimately, three feature representation methods exhibiting the most stable performance were selected: ESM N-terminal mean (average ESM features of the N-terminal region), DR, and SC-PseAAC. These were used as input features for the model (Fig 4B). During model training, we utilized a data loader to dynamically load data for different tasks, assigning task-specific labels. Samples corresponding to the current task were labelled as 1, while others were labelled as 0. This task partitioning approach allowed the model to effectively capture distinguishing information between tasks while facilitating the learning of shared features. The model architecture consists of the following four stages:

(1) Feature combination

The ESM N-terminal mean, DR, and SC-PseAAC are concatenated and integrated to form a unified feature representation. This representation combines local sequence information (such as N-terminal specificity), dipeptide repeat patterns, and sequence-related information.

(2) Shared backbone

The unified representation is fed into a shared backbone network for deep feature learning. This network consists of two fully connected (Linear) layers, each followed by an activation function (ReLU) and a Dropout layer (with a dropout probability of 0.2). This shared backbone network effectively captures common features across different tasks, enhancing the generalization ability of the model and reducing the number of training parameters.

(3) Task-specific heads

Features obtained from the shared backbone network are separately fed into five task-specific classification heads, each corresponding to one effector type (T1/2/3/4/6SE). Each task head is an independent linear classifier that outputs classification probabilities for the respective task.

(4) Multi-task training process

During training, each data batch is divided according to the task type. For each task, a task specific mask is created to select the corresponding sample features and labels. Losses are calculated separately using the BCEWithLogitsLoss function, then summed up for backpropagation. This training approach ensures that the model learns common features while focusing on task-specific differences, thus improving the overall classification performance.

## Supporting information

S1 Text Supplemental methods, analyses, and evaluation of the TXSelect model. This document provides additional methodological details, including feature visualization analyses, hyperparameter optimization procedures, feature attribution analyses, and homology-controlled model generalization evaluations.
(DOCX)

S1 Fig UMAP visualization of selected features used in TXSelect. (TIF)

S2 Fig Hyperparameter tuning of TXSelect and its impact on validation and test performance. (TIF)

S3 Fig Feature attribution heatmap for T1SE task. (TIF)

S4 Fig Feature attribution heatmap for T2SE task. (TIF)

S5 Fig Feature attribution heatmap for T3SE task. (TIF)

S6 Fig Feature attribution heatmap for T4SE task. (TIF)

S7 Fig Feature attribution heatmap for T6SE task. (TIF)

S8 Fig Top 20 feature importance for T1SE task based on SHAP values. (TIF)

S9 Fig Top 20 feature importance for T2SE task based on SHAP values. (TIF)

S10 Fig Top 20 feature importance for T3SE task based on SHAP values. (TIF)

S11 Fig Top 20 feature importance for T4SE task based on SHAP values. (TIF)

S12 Fig Top 20 feature importance for T6SE task based on SHAP values. (TIF)

S13 Fig Training of TXSelect under 50% sequence identity cut-off. (TIF)

S14 Fig Performance heatmaps of TXSelect under 50% sequence identity cut-off. (TIF)
