# Supplementary material for: TXSelect: A multi-task learning model to identify secretory effectors
Source: PLoS Comput Biol. 2025 Nov 6;21(11):e1013677. doi: 10.1371/journal.pcbi.1013677 (PMC12591437; doi:10.1371/journal.pcbi.1013677)
Supplement: S1 Text — This document provides additional methodological details, including feature visualization analyses, hyperparameter optimization procedures, feature attribution analyses, and homology-controlled model generalization evaluations. (DOCX) [file pcbi.1013677.s001.docx]

#### **Supplemental information**

**TXSelect: A multi-task learning model to identify secretory effectors**

#### **1. Visualization analysis of selected features**

To intuitively understand and verify the contributions of the selected features (ESM n-terminal mean, DR, and SC-PseAAC) to multi-task classification performance, we employed UMAP [4] to conduct a two-dimensional visual analysis of features from different types of effectors in the training and validation sets (Figure S1).

Figures S1A–B illustrate the visualization results of the ESM n-terminal mean feature for the T1/2SE and T3/4/6SE subtasks. It can be observed that ESM n-terminal mean effectively differentiates between different types of effectors, particularly demonstrating clear distinctions in the T1/2SE subtask, which aligns well with the previously described classification performance results [5]. Figures S1C–D present the UMAP dimensionality reduction results for the DR feature, similarly, showing relatively clear cluster separations for different subtasks, indicating that the DR feature also has significant discriminative capability across different effector types. Figures S1E–F display the visualization outcomes for the SC-PseAAC feature, which exhibit somewhat complex cluster distributions within the T3/4/6SE subtask [6, 7].

Overall, the UMAP visualization results further substantiate the feature performance trends observed in previous classification tasks, demonstrating that the combination of ESM n-terminal mean, DR, and SC-PseAAC features within the TXSelect multi-task framework possesses excellent cluster separation effects and robust generalization capability.

#### **2. Hyperparameter optimization**

To further optimize model performance and enhance predictive generalization, this study systematically optimized five key hyperparameters involved in the multi-task model [1]. We employed a controlled variable approach, where one hyperparameter was tuned at a time while keeping other parameters at their default settings, ensuring the comparability and clarity of experimental results. The specific optimization procedures were as follows.

(1) Batch size optimization (Figure S2A). We tested three batch sizes—32, 64 (TXSelect), and 128—using the F1-score as the performance evaluation criterion [2].

(2) Hidden dimensions optimization (Figure S2B). We evaluated three different combinations of hidden dimensions: [512, 256, 128], [256, 128] (TXSelect), and [128, 64], assessing the impact of each dimensional combination on model generalization performance.

(3) Optimizer selection (Figure S2C). We systematically compared four common optimizers: Adam (TXSelect), SGD, AdamW, and RMSprop, to identify the optimizer most suitable for the context of this study.

(4) Learning rate optimization (Figure S2D)

We conducted detailed tests and comparisons across four different learning rates: 1e-2, 1e-3 (TXSelect), 1e-4, and 1e-5, to identify the optimal learning rate.

(5) Dropout probability optimization (Figure S2E)

We systematically evaluated the effect of five dropout probabilities—0.1, 0.2 (TXSelect), 0.3, 0.4, and 0.5—on model performance.

We used the validation set F1-score as the critical criterion for hyperparameter tuning, supplemented by test set performance for auxiliary confirmation [3]. After the systematic optimization described above, the final optimal hyperparameter combination was determined as follows: Batch size = 64, Hidden dimensions = [256, 128], Optimizer = Adam, Learning rate = 1e-3, Dropout probability = 0.2. This combination exhibited the best and most stable performance on both validation and test sets and was therefore selected as the final hyperparameter configuration for the multi-task model in this study.

**3.** **SHAP feature attribution heatmap and feature importance analysis**

To further enhance the interpretability of the model and reveal the roles of different features in classification decisions across tasks, we conducted a systematic feature-level attribution analysis of the multi-task neural network model using the SHAP method. The relevant results are as follows:

**3.1 Feature attribution heatmap**

For each type of effector protein (T1SE, T2SE, T3SE, T4SE, T6SE), we plotted attribution heatmaps of the top 30 most important features across all test samples (see Figure S3–S7). The heatmap visually presents the specific contribution of each feature to different sample predictions with color intensity (red for positive attribution, blue for negative attribution). Samples are sorted by the model’s predicted probability, making it convenient to observe how feature attributions change along with the classification tendency of the samples. From the heatmap results, the attribution patterns of key features vary significantly across tasks, and some features exhibit markedly different contribution directions in positive and negative samples.

**3.2 Feature importance bar plot**

We calculated the mean absolute SHAP values of the top 20 features for each task and presented them as bar plots (see Figure S8–S12). This ranking clearly shows the most influential features for each task, facilitating comparison of the relative importance of different feature types including ESM sequence embedding, distance-based residue descriptors (DR), and SC-PseAAC in classification.

The results show that certain ESM N-terminal mean features and DR features repeatedly appear at the top across multiple tasks, indicating that these representations are generally applicable for discriminating effector protein classes. Meanwhile, the distribution of feature importance differs significantly among tasks, reflecting the model’s ability to capture task-specific discriminative information. The specific conclusions are as follows:

**(1) High frequency important features across tasks**

ESM N terminal mean feature 143 and DR feature 593 frequently appear among the top two or three features in all five tasks, with high mean absolute attribution values (mean absolutely importance): T1SE: 3.83 (feature 143) / 3.71 (feature 593); T2SE: 4.51 (feature 143) / 4.66 (feature 593); T3SE: 2.31 (feature 143) / 2.27 (feature 593); T4SE: 3.44 (feature 143) / 3.47 (feature 593); T6SE: 3.39 (feature 143) / 3.47 (feature 593). This indicates that ESM N-terminal sequence embedding features and DR structure-related features are the core features for distinguishing different effector protein types, with strong generalization and discriminative power in the multi-task learning framework. DR feature 419 also ranks among the top five in all categories.

**(2) Task-specific discriminative features**

Some features rank highly in specific classes but are lower in others. For example, in T2SE, DR feature 363 (4.21), DR feature 419 (4.13), and DR feature 289 (4.13) show certain class-specific contributions. In T3SE and T4SE, certain ESM features (such as ESM N terminal mean feature 215, ESM N terminal mean feature 232) and DR features are both among the top ten, indicating that the model can adaptively extract the most discriminative feature subsets for different tasks.

**(3) Importance of traditional descriptors for SC-PseAAC**

SC-PseAAC feature 0 is relatively stable in its ranking among all classes, always within the top 20. Although not as prominent as ESM and DR features, it still plays a supplementary role in discrimination, suggesting that traditional features have supplementary value in a multimodal feature combination framework.

**4. Homology-Controlled Evaluation of Model Generalization**

To objectively evaluate the cross-homology generalization ability of TXSelect and further address the issue of potential homology leakage, we conducted a supplementary experiment using a more stringent homology-controlled setting. Specifically, all effector protein sequences were reclustered using CD-HIT with a 50% sequence identity threshold, ensuring that no pair of sequences in the training, validation, and test sets exceeded this cutoff. This partitioning strategy is more rigorous than the previously used 70% threshold. The feature extraction pipeline, model architecture, and training procedures remained consistent with those described in the main text. The training process and the heatmaps of the model’s performance on the validation and test sets are shown in Figure S13 and Figure S14. Under this homology-controlled scenario, TXSelect achieved an F1 score of 0.88 on the validation set and 0.86 on the test set, which is highly comparable to the results obtained with the 70% cutoff (validation F1 = 0.867, test F1 = 0.8645). These results demonstrate that TXSelect retains strong generalization performance even when evaluated under stricter sequence identity constraints. The minimal change in F1 scores indicates that the model is not overly dependent on homologous sequence similarities but rather captures informative and robust features that support effective classification of distant homologues and potentially novel effector proteins.

**Reference**

1. Claesen, M. and B. De Moor, *Hyperparameter search in machine learning.* arXiv preprint arXiv:1502.02127, 2015.

2. Keskar, N.S., et al., *On large-batch training for deep learning: Generalization gap and sharp minima.* arXiv preprint arXiv:1609.04836, 2016.

3. Friedman, J., *The elements of statistical learning: Data mining, inference, and prediction.* (No Title), 2009.

4. McInnes, L., J. Healy, and J. Melville, *Umap: Uniform manifold approximation and projection for dimension reduction.* arXiv preprint arXiv:1802.03426, 2018.

5. Creagh, A.P., et al., *Interpretable deep learning for the remote characterisation of ambulation in multiple sclerosis using smartphones.* Scientific Reports, 2021. **11**(1): p. 14301.

6. Galán, J.E. and A. Collmer, *Type III secretion machines: bacterial devices for protein delivery into host cells.* Science, 1999. **284**(5418): p. 1322-1328.

7. Cascales, E. and P.J. Christie, *The versatile bacterial type IV secretion systems.* Nature Reviews Microbiology, 2003. **1**(2): p. 137-149.

**Supporting Information Figure Legends**

**Figure S1*.* UMAP visualization of selected features used in TXSelect.**

UMAP projections of the three selected features (ESM n-terminal mean, DR, and SC-PseAAC) on training and validation sets for different effector subsets. (A–B) ESM n-terminal mean for T1/2SE and T3/4/6SE. (C–D) DR for T1/2SE and T3/4/6SE. (E–F) SC-PseAAC for T1/2SE and T3/4/6SE.

**Figure S2. Hyperparameter tuning of TXSelect and its impact on validation and test performance.**

(A) Batch size (32, 64, 128). (B) Hidden dimension configurations ([512, 256, 128], [256, 128], [128, 64]). (C) Optimizers (Adam, SGD, AdamW, RMSprop). (D) Learning rates (1e-2, 1e-3, 1e-4, 1e-5). (E) Dropout probabilities (0.1, 0.2, 0.3, 0.4, 0.5).

**Figure S3. Feature attribution heatmap for T1SE task.**

The heatmap displays the SHAP values (attribution values) of the top 30 most important features across all test samples in the T1SE task. Samples are sorted by the model's predicted probability, and color intensity indicates the contribution direction and magnitude (red: positive, blue: negative).

**Figure S4. Feature attribution heatmap for T2SE task.**

The heatmap displays the SHAP values (attribution values) of the top 30 most important features across all test samples in the T2SE task. Samples are sorted by the model's predicted probability, and color intensity indicates the contribution direction and magnitude (red: positive, blue: negative).

**Figure S5. Feature attribution heatmap for T3SE task.**

The heatmap displays the SHAP values (attribution values) of the top 30 most important features across all test samples in the T3SE task. Samples are sorted by the model's predicted probability, and color intensity indicates the contribution direction and magnitude (red: positive, blue: negative).

**Figure S6. Feature attribution heatmap for T4SE task.**

The heatmap displays the SHAP values (attribution values) of the top 30 most important features across all test samples in the T4SE task. Samples are sorted by the model's predicted probability, and color intensity indicates the contribution direction and magnitude (red: positive, blue: negative).

**Figure S7. Feature attribution heatmap for T6SE task.**

The heatmap displays the SHAP values (attribution values) of the top 30 most important features across all test samples in the T6SE task. Samples are sorted by the model's predicted probability, and color intensity indicates the contribution direction and magnitude (red: positive, blue: negative).

**Figure S8. Top 20 feature importance for T1SE task based on SHAP values.**

The bar plot shows the mean absolute SHAP values of the top 20 most important features for the T1SE task, reflecting their contributions to the classification decisions.

**Figure S9. Top 20 feature importance for T2SE task based on SHAP values.**

The bar plot shows the mean absolute SHAP values of the top 20 most important features for the T2SE task, reflecting their contributions to the classification decisions.

**Figure S10. Top 20 feature importance for T3SE task based on SHAP values.**

The bar plot shows the mean absolute SHAP values of the top 20 most important features for the T3SE task, reflecting their contributions to the classification decisions.

**Figure S11. Top 20 feature importance for T4SE task based on SHAP values.**

The bar plot shows the mean absolute SHAP values of the top 20 most important features for the T4SE task, reflecting their contributions to the classification decisions.

**Figure S12. Top 20 feature importance for T6SE task based on SHAP values.**

The bar plot shows the mean absolute SHAP values of the top 20 most important features for the T6SE task, reflecting their contributions to the classification decisions.

**Figure S13. Training of TXSelect under 50% sequence identity cut-off.**

The plot shows the training loss and the F1 scores of each effector type (T1SE, T2SE, T3SE, T4SE, T6SE) over 500 epochs when the dataset is clustered at 50% sequence identity using CD-HIT.

**Figure S14. Performance heatmaps of TXSelect under 50% sequence identity cut-off.**

(A) Classification report heatmap for the validation dataset. (B) Classification report heatmap for the test dataset. Each cell represents the AUC, F1 score, precision, and recall for each effector class.
