# Supplementary figures and images for: TXSelect: A multi-task learning model to identify secretory effectors
Source: PLoS Comput Biol. 2025 Nov 6;21(11):e1013677. doi: 10.1371/journal.pcbi.1013677 (PMC12591437; doi:10.1371/journal.pcbi.1013677)

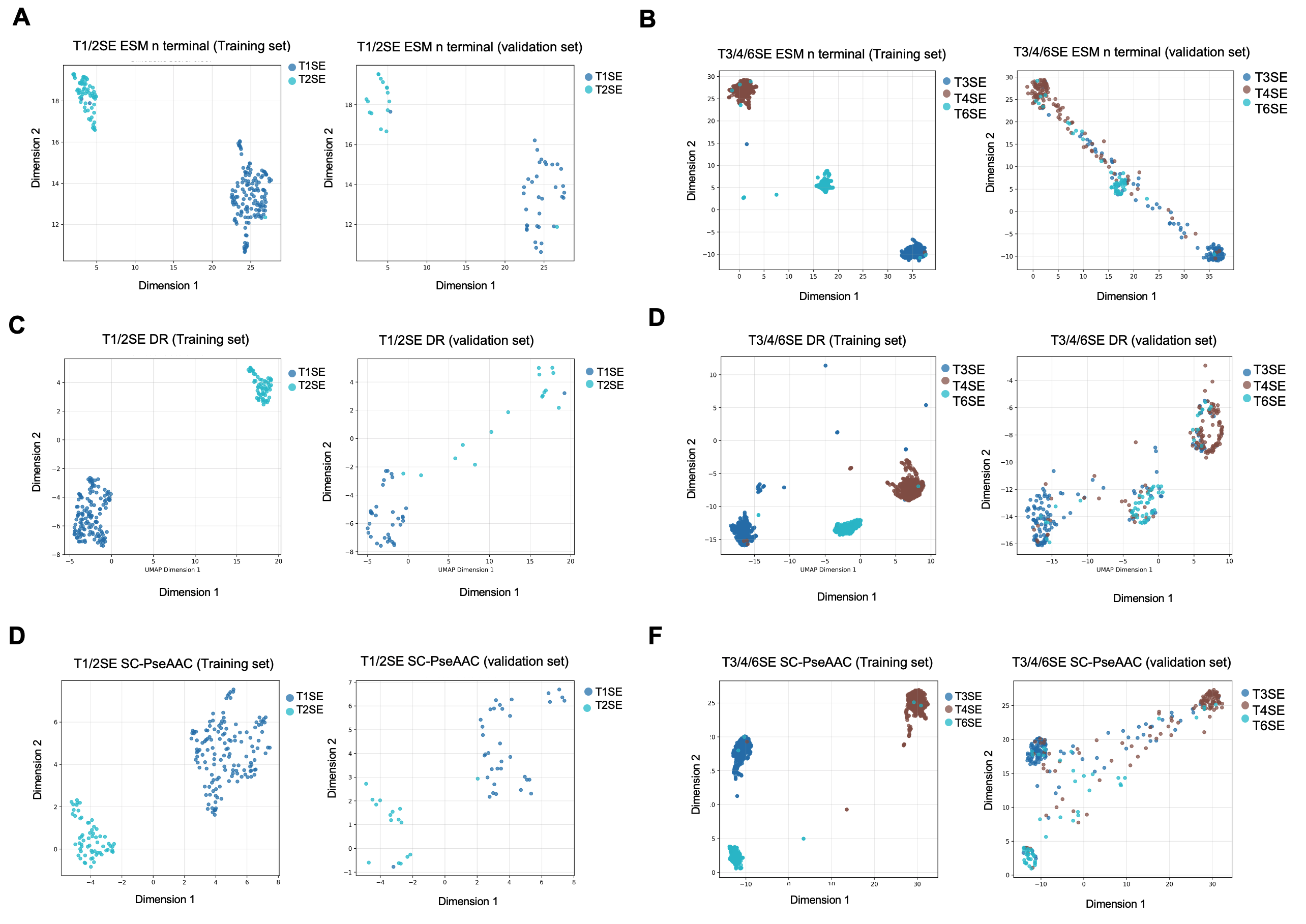

Supplement: S1 Fig — (TIF) [file pcbi.1013677.s002.tif]

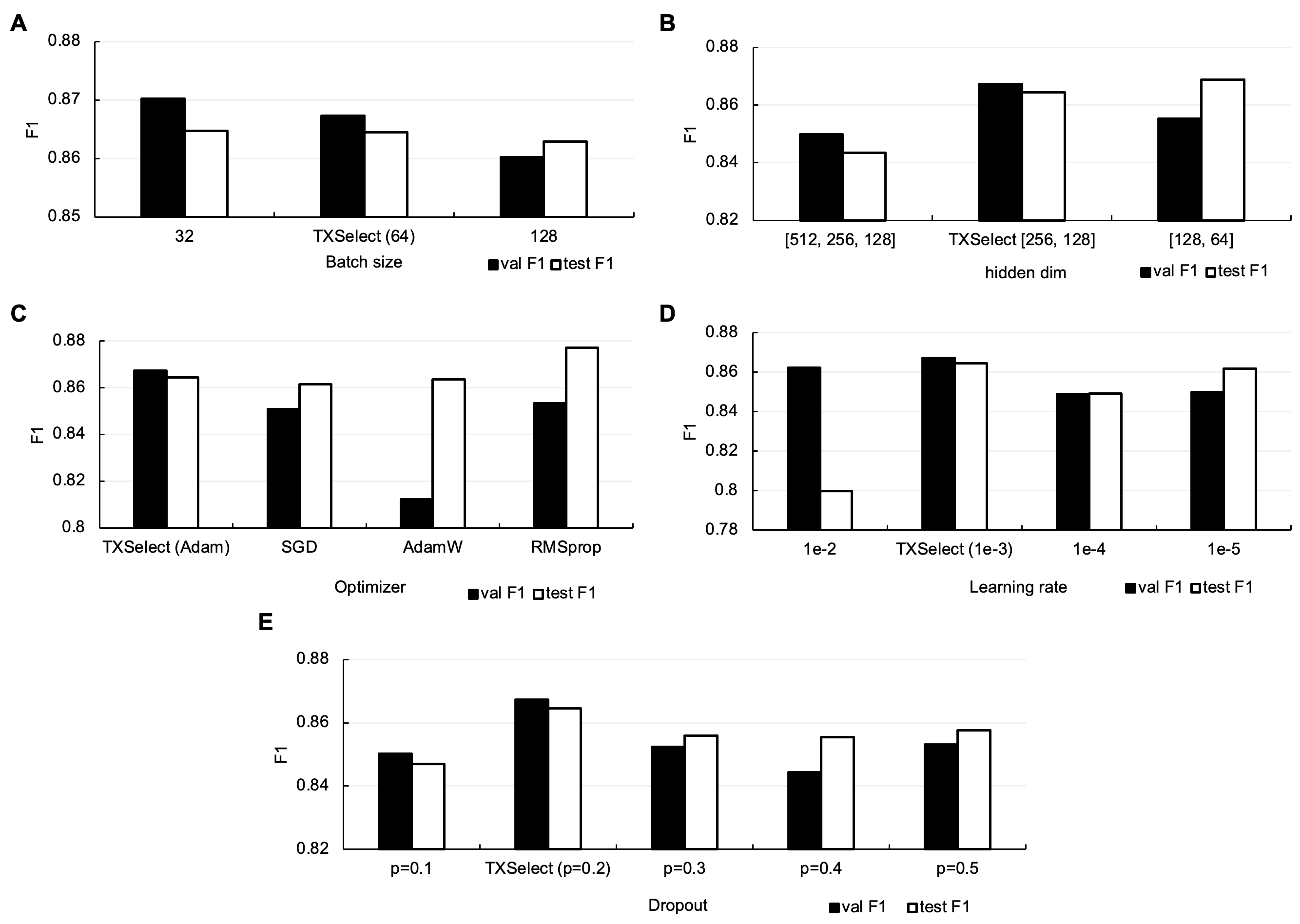

Supplement: S2 Fig — (TIF) [file pcbi.1013677.s003.tif]

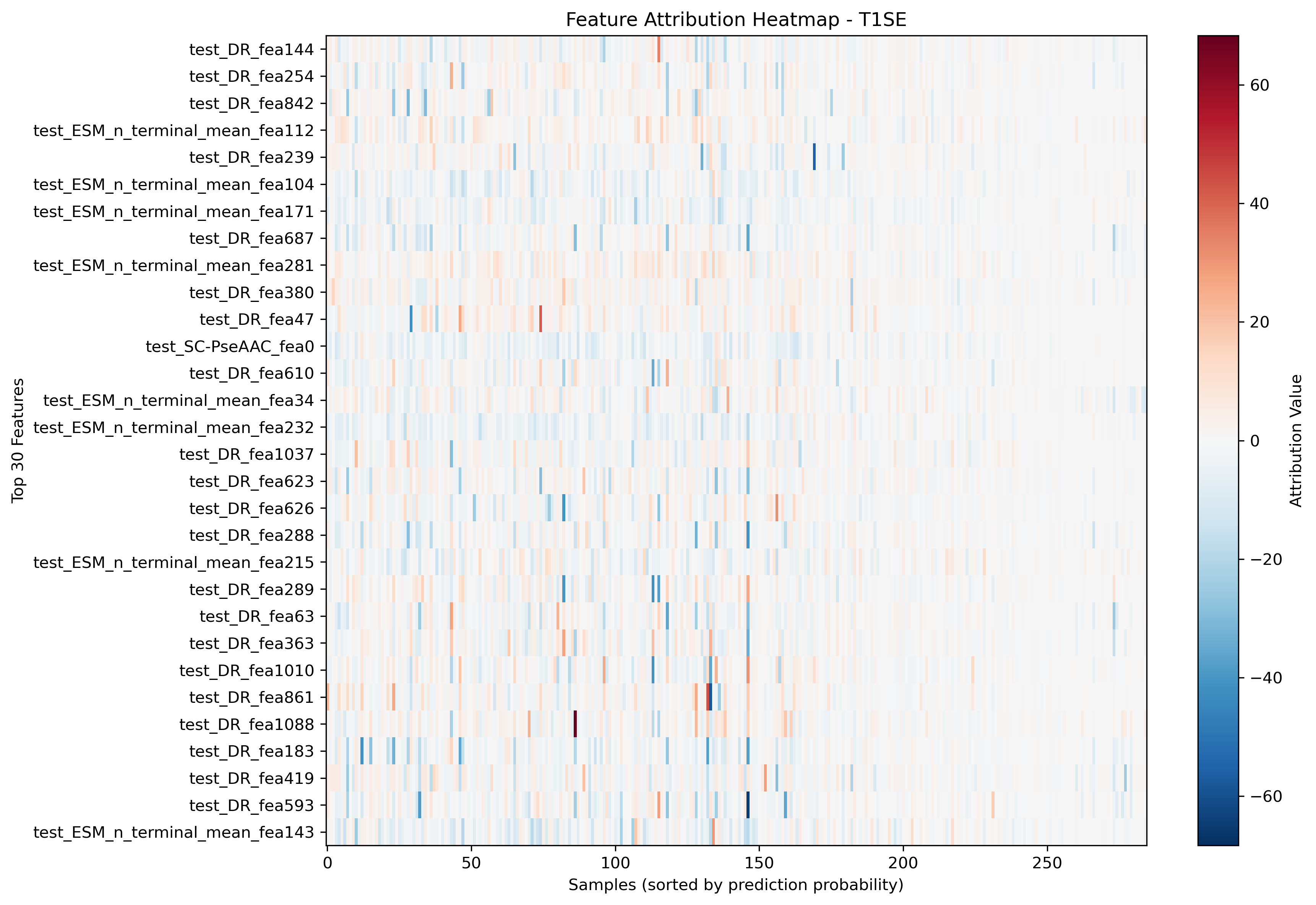

Supplement: S3 Fig — (TIF) [file pcbi.1013677.s004.tif]

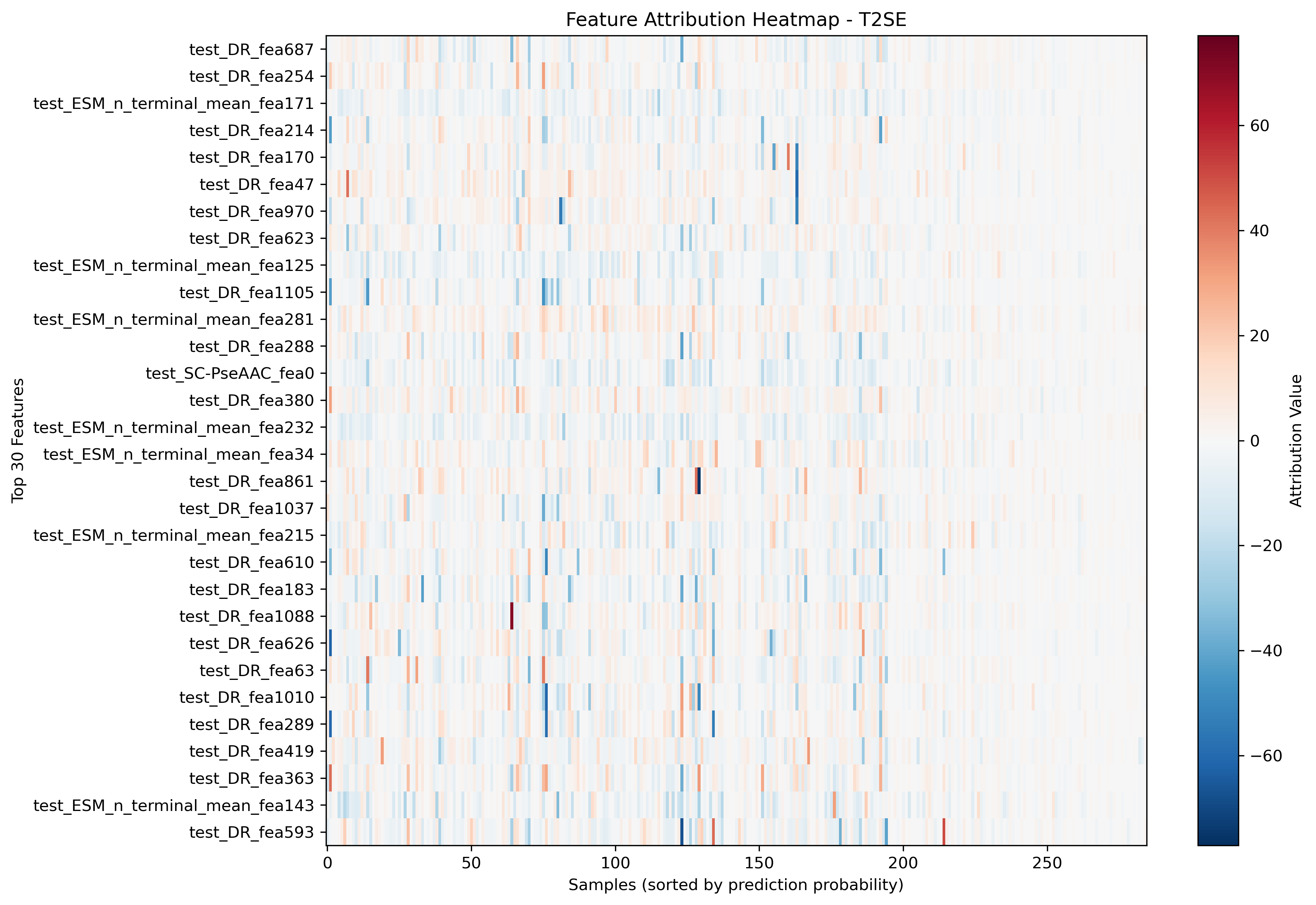

Supplement: S4 Fig — (TIF) [file pcbi.1013677.s005.tif]

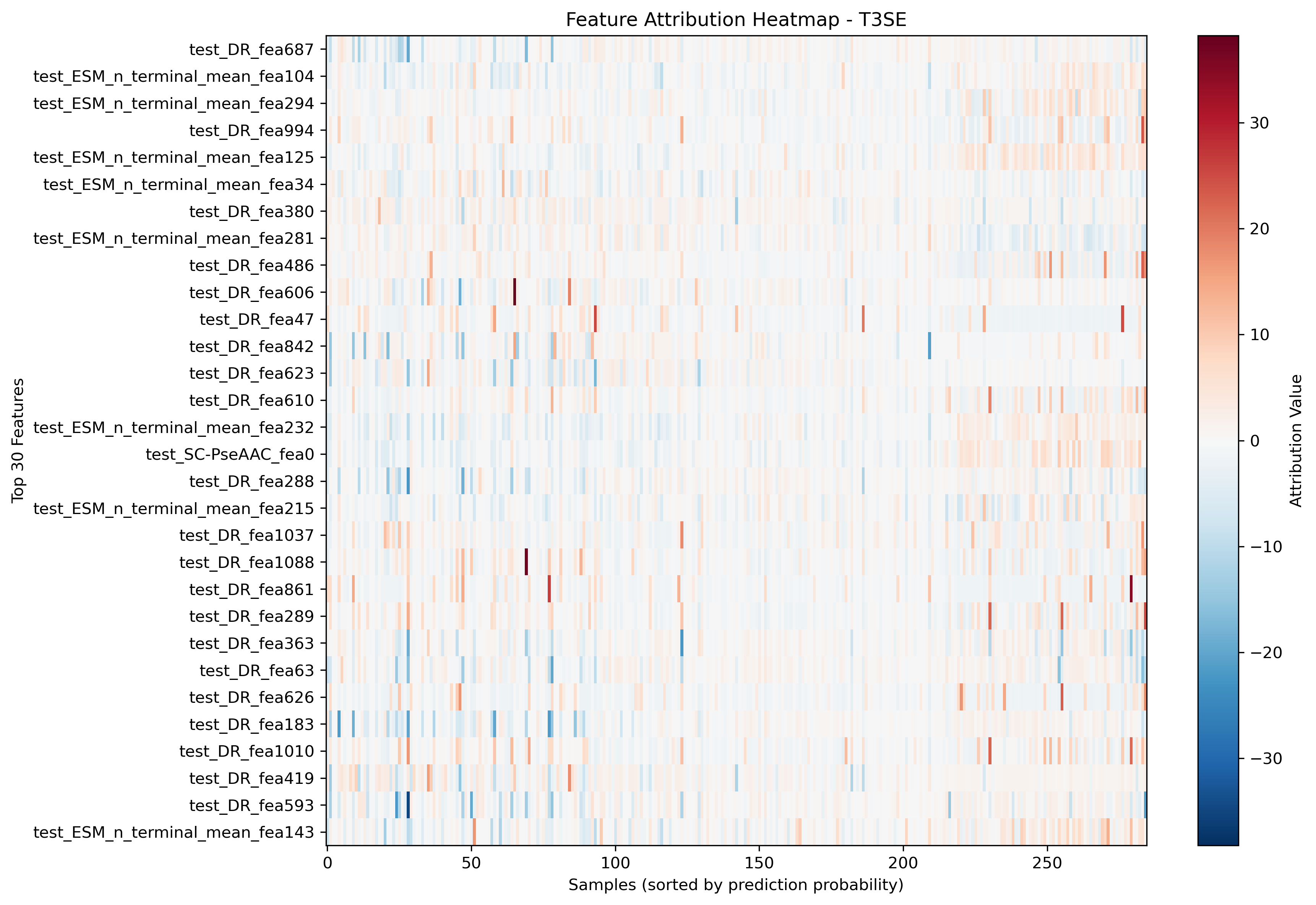

Supplement: S5 Fig — (TIF) [file pcbi.1013677.s006.tif]

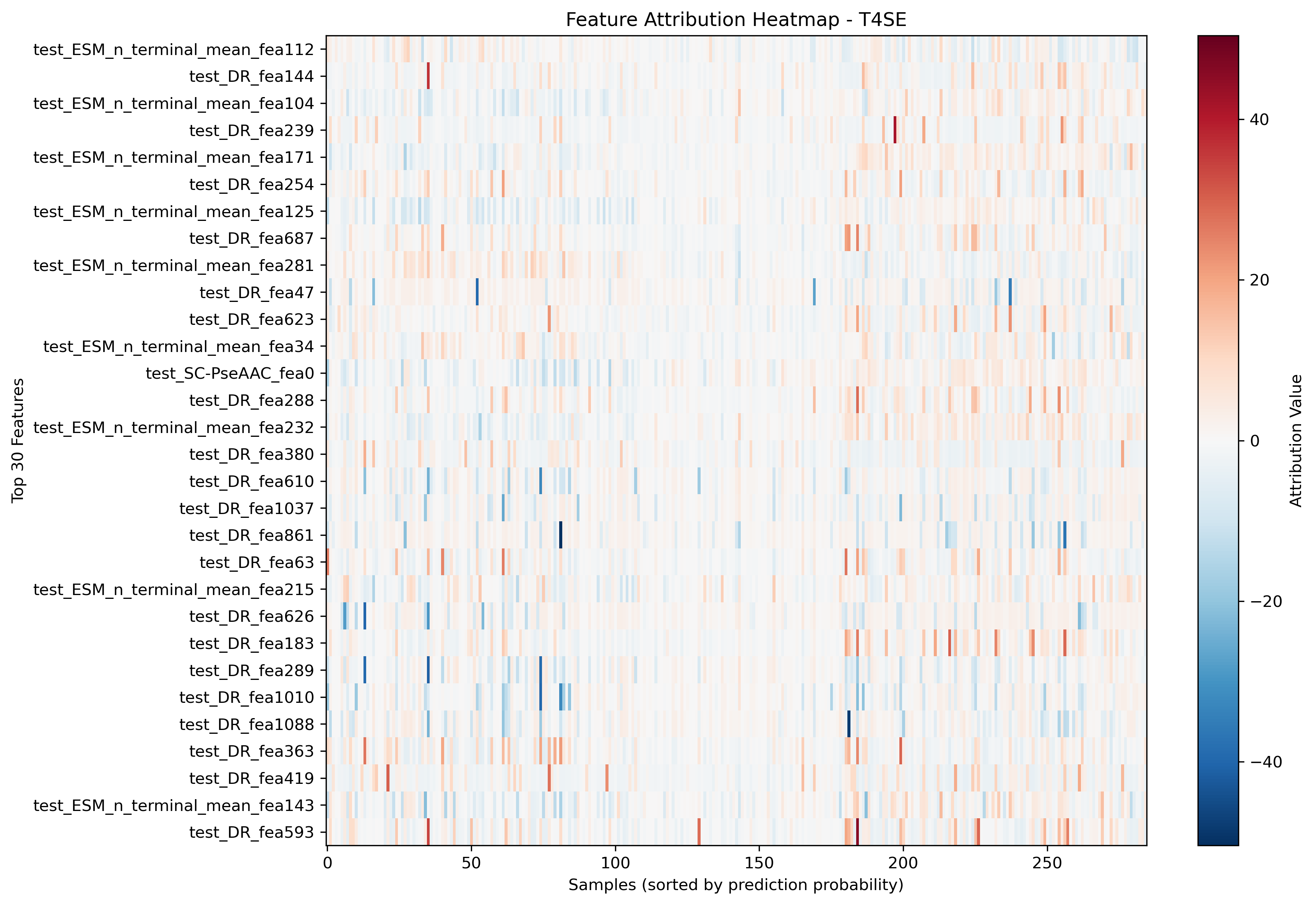

Supplement: S6 Fig — (TIF) [file pcbi.1013677.s007.tif]

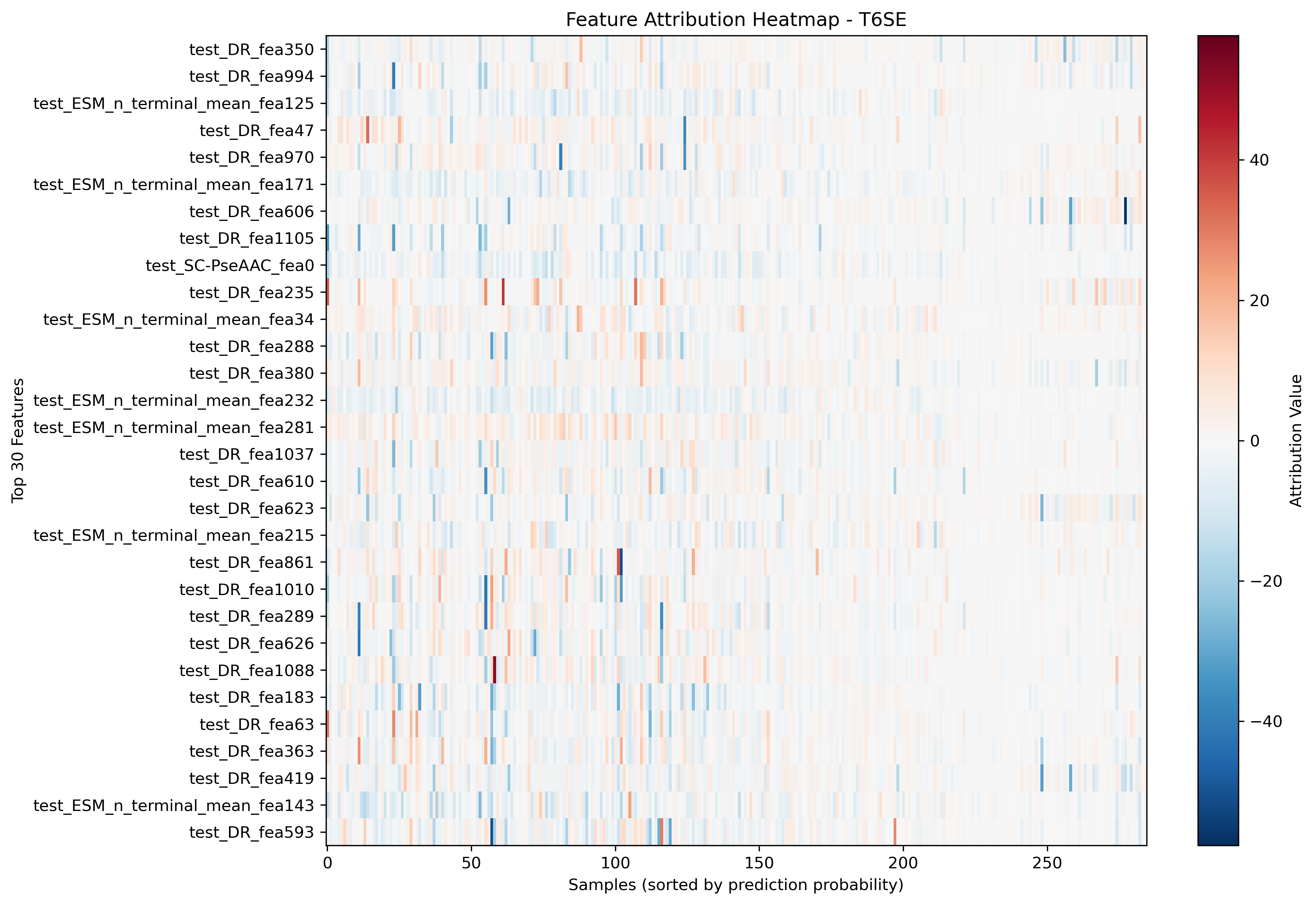

Supplement: S7 Fig — (TIF) [file pcbi.1013677.s008.tif]

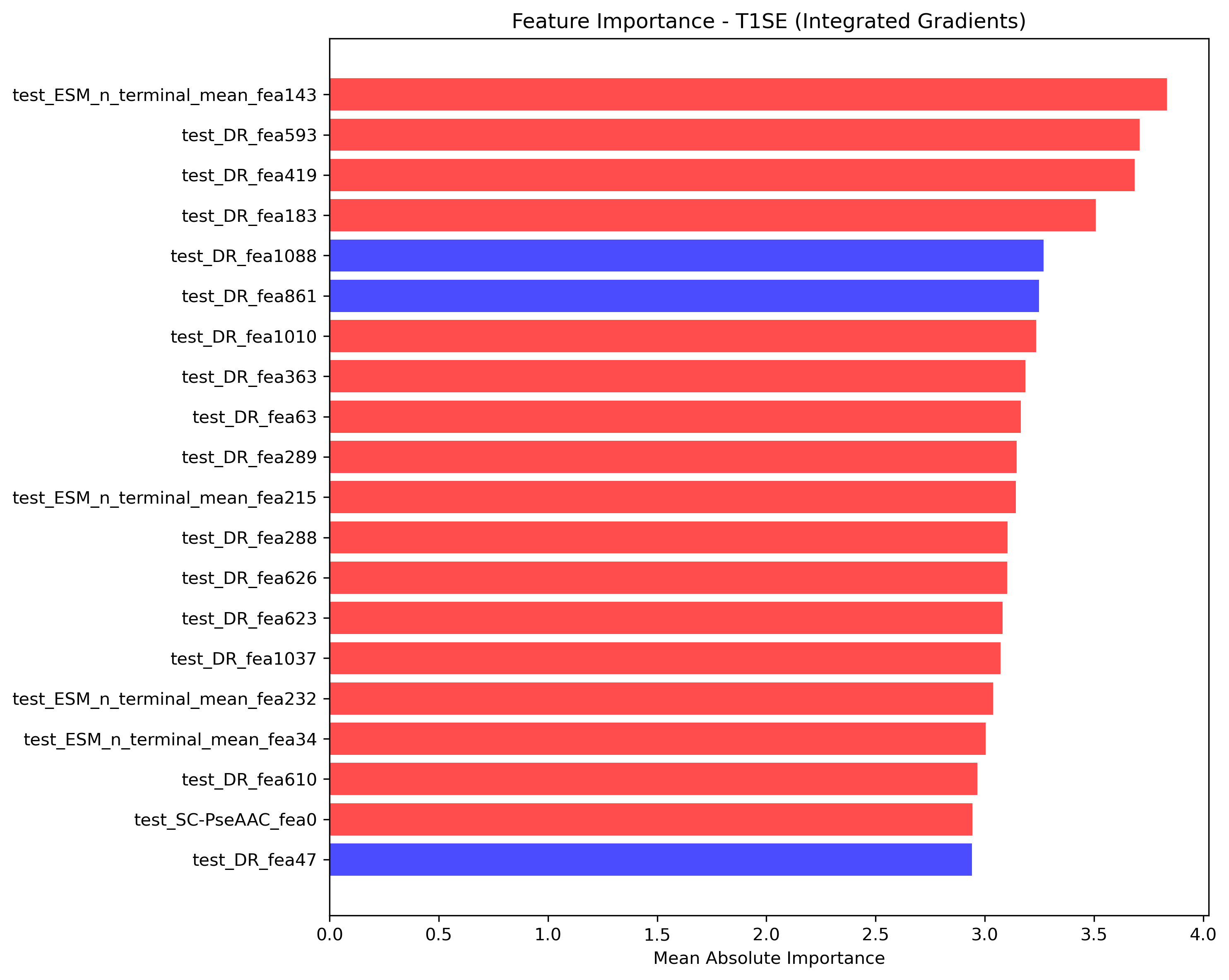

Supplement: S8 Fig — (TIF) [file pcbi.1013677.s009.tif]

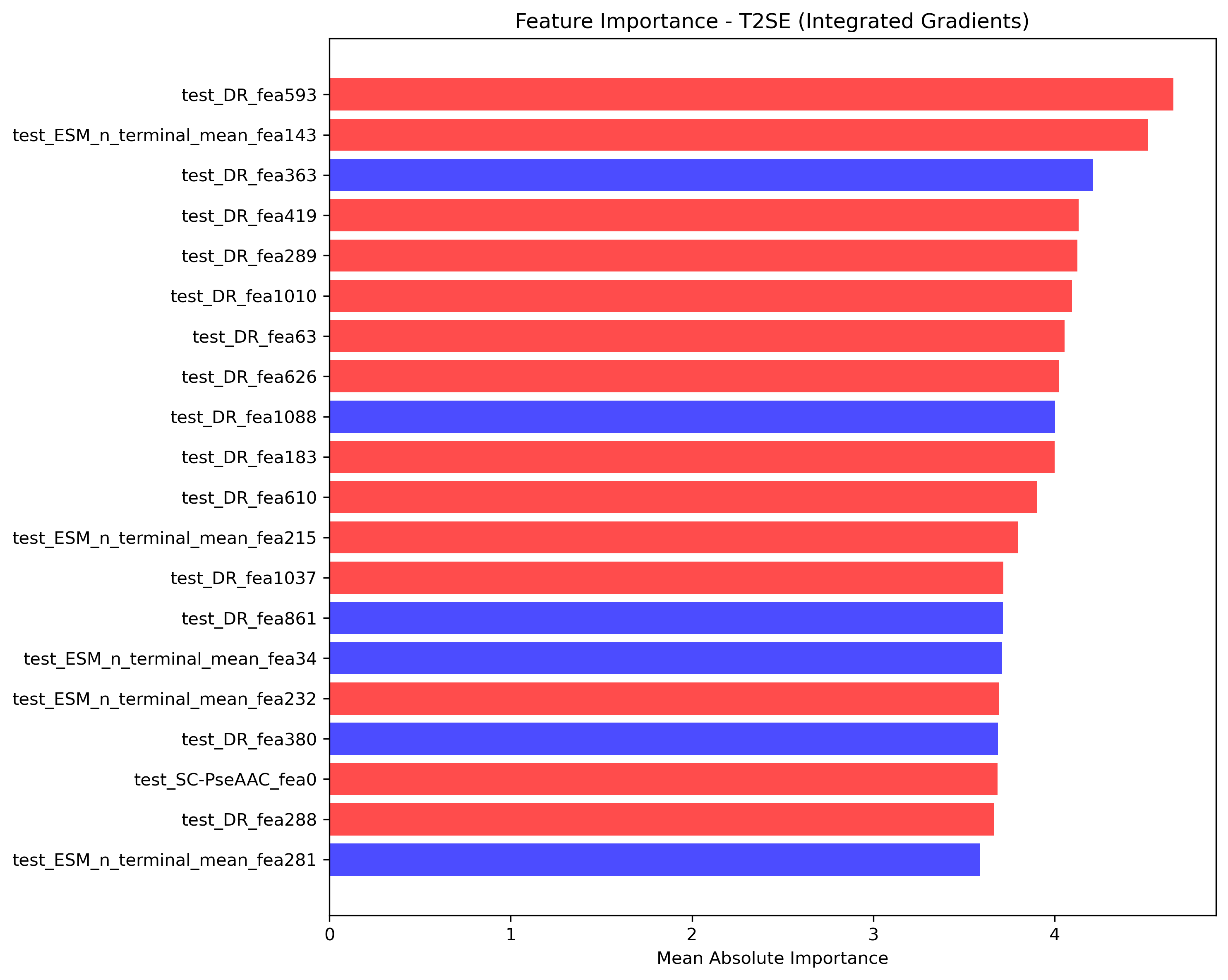

Supplement: S9 Fig — (TIF) [file pcbi.1013677.s010.tif]

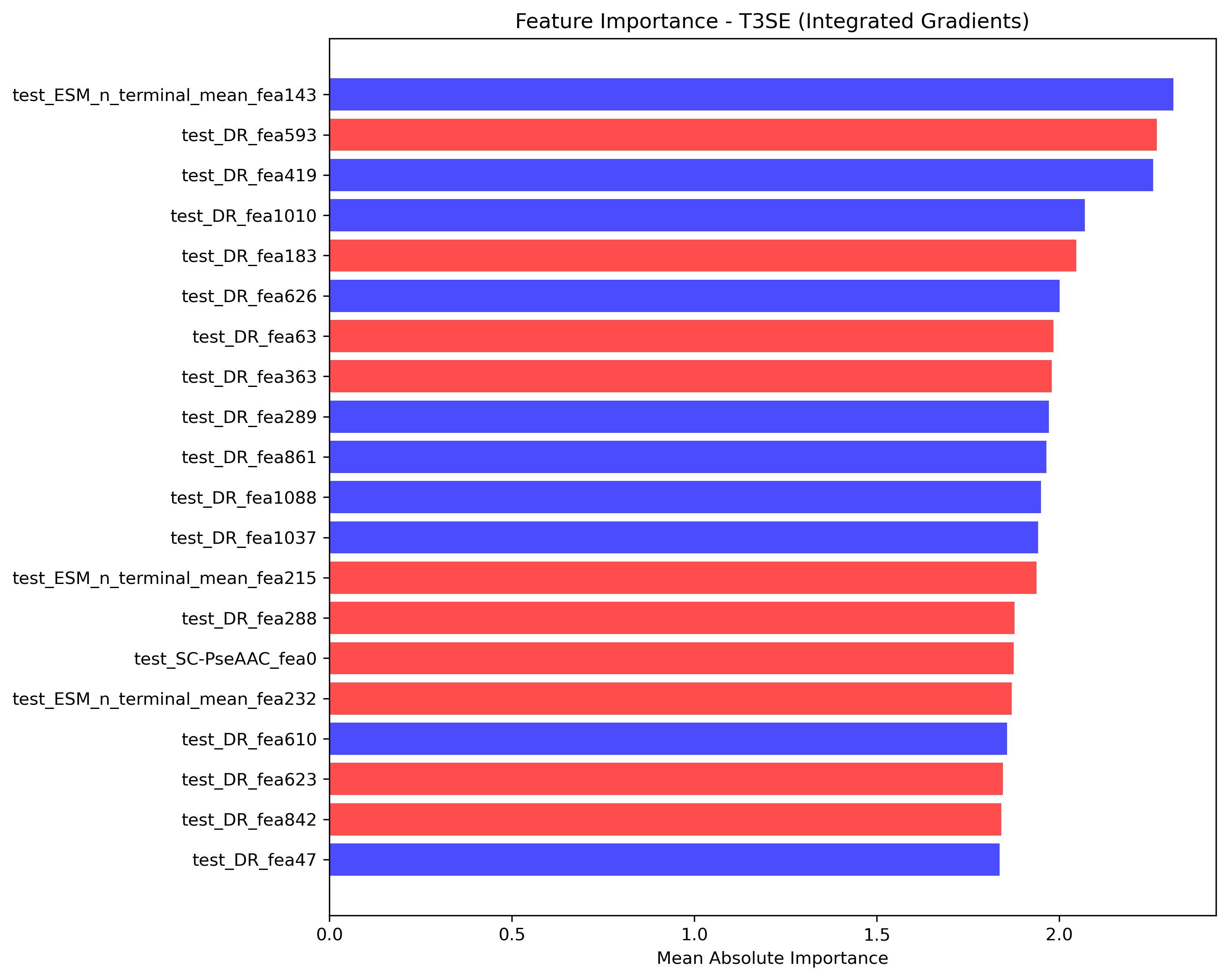

Supplement: S10 Fig — (TIF) [file pcbi.1013677.s011.tif]

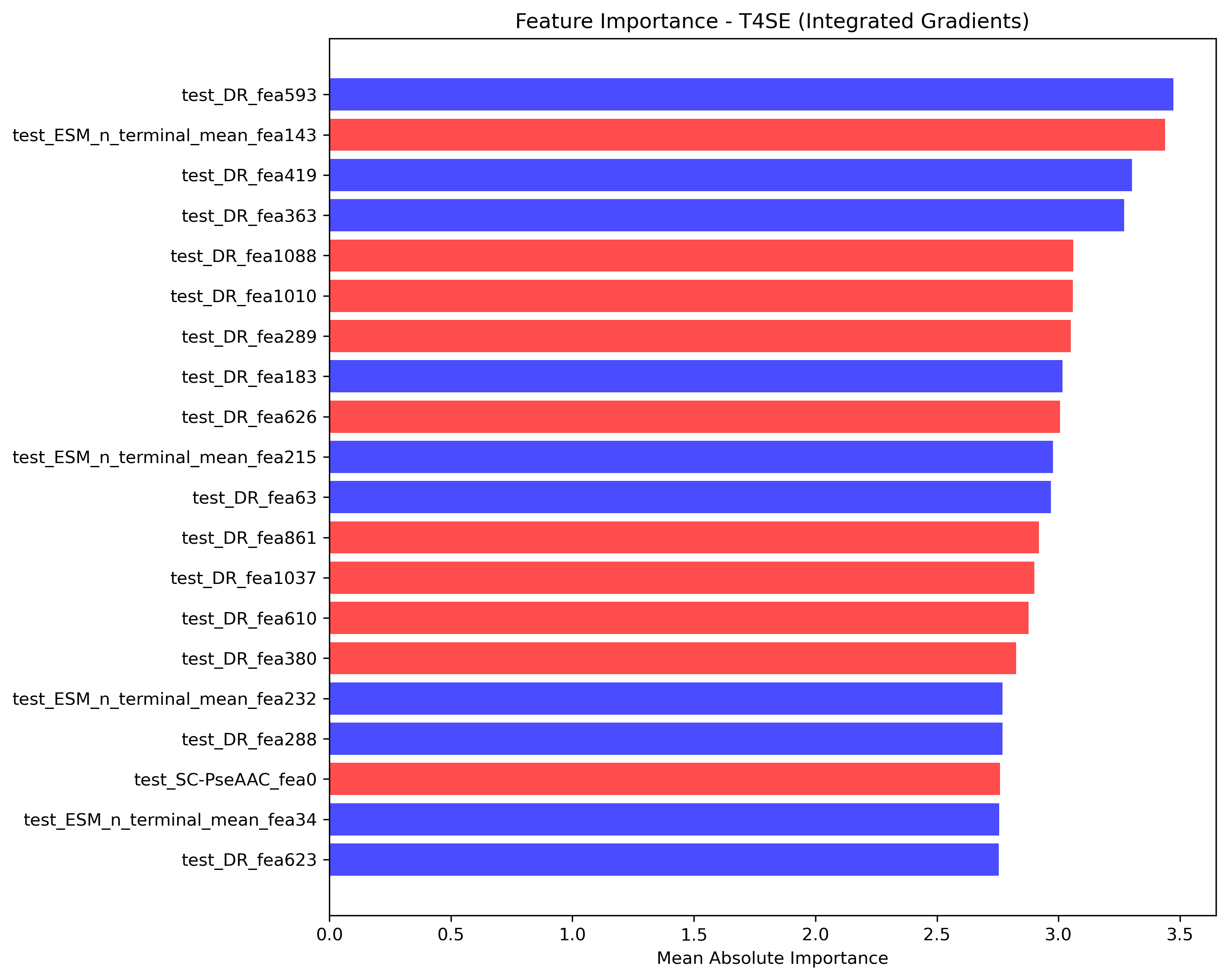

Supplement: S11 Fig — (TIF) [file pcbi.1013677.s012.tif]

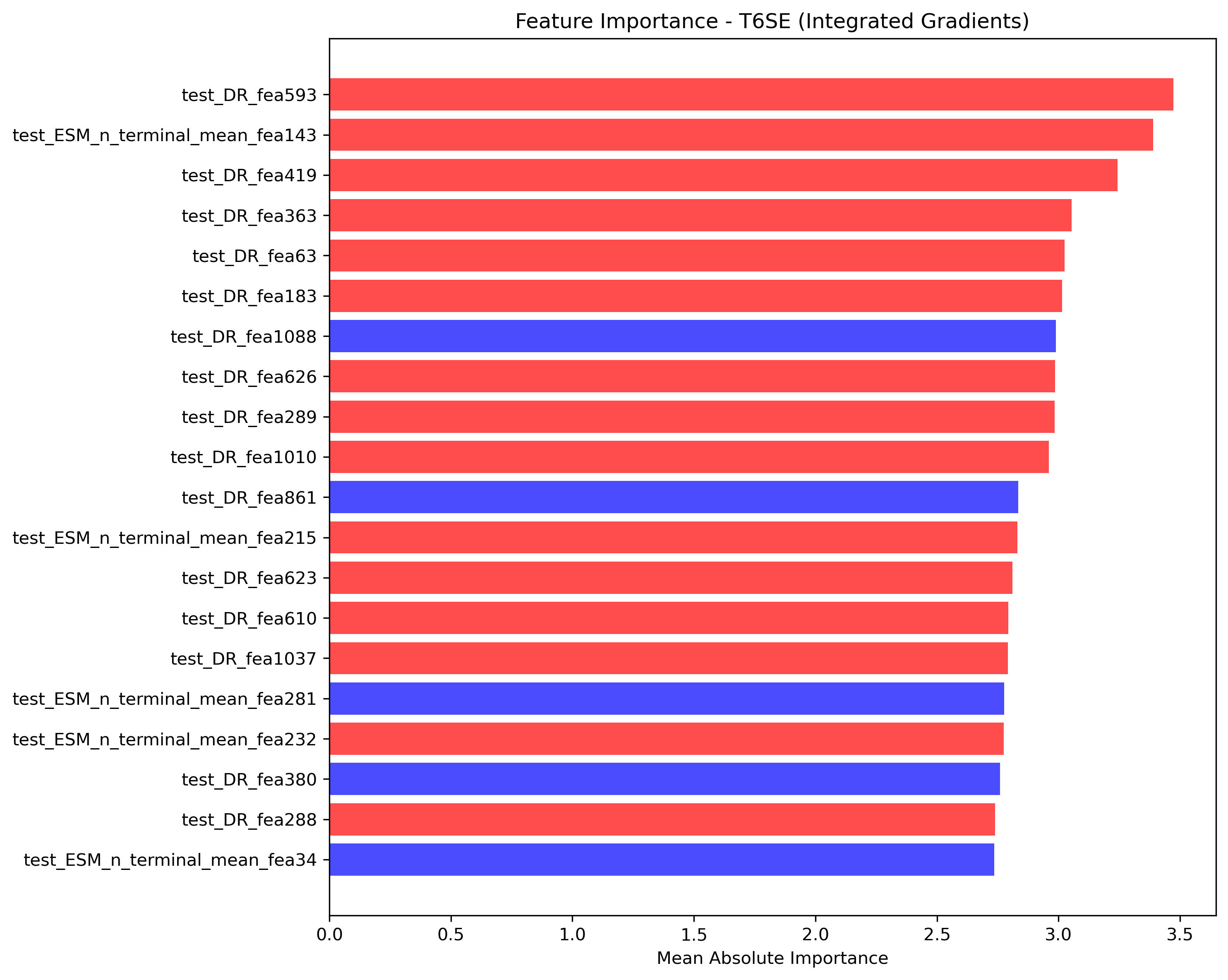

Supplement: S12 Fig — (TIF) [file pcbi.1013677.s013.tif]

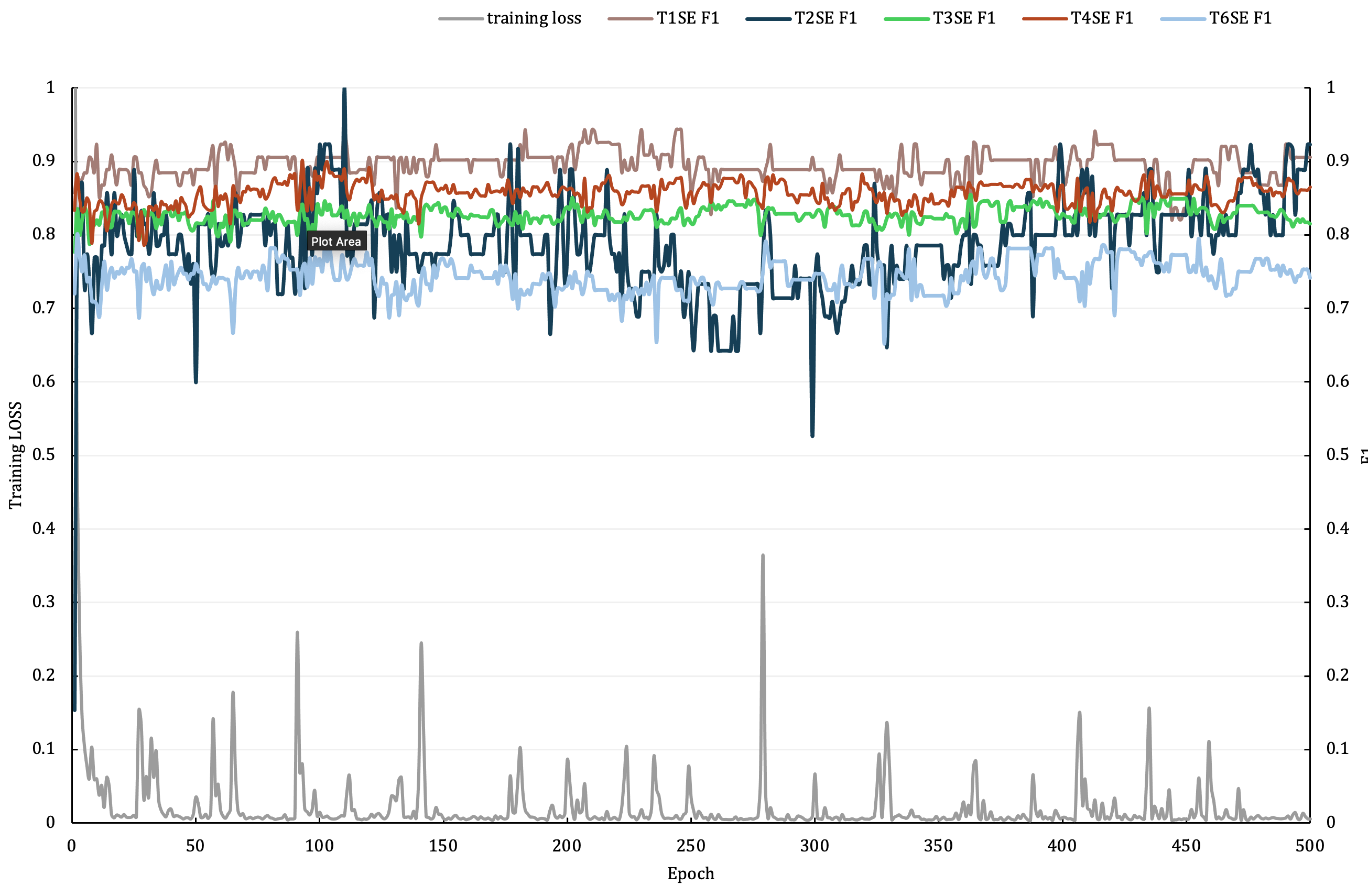

Supplement: S13 Fig — (TIF) [file pcbi.1013677.s014.tif]

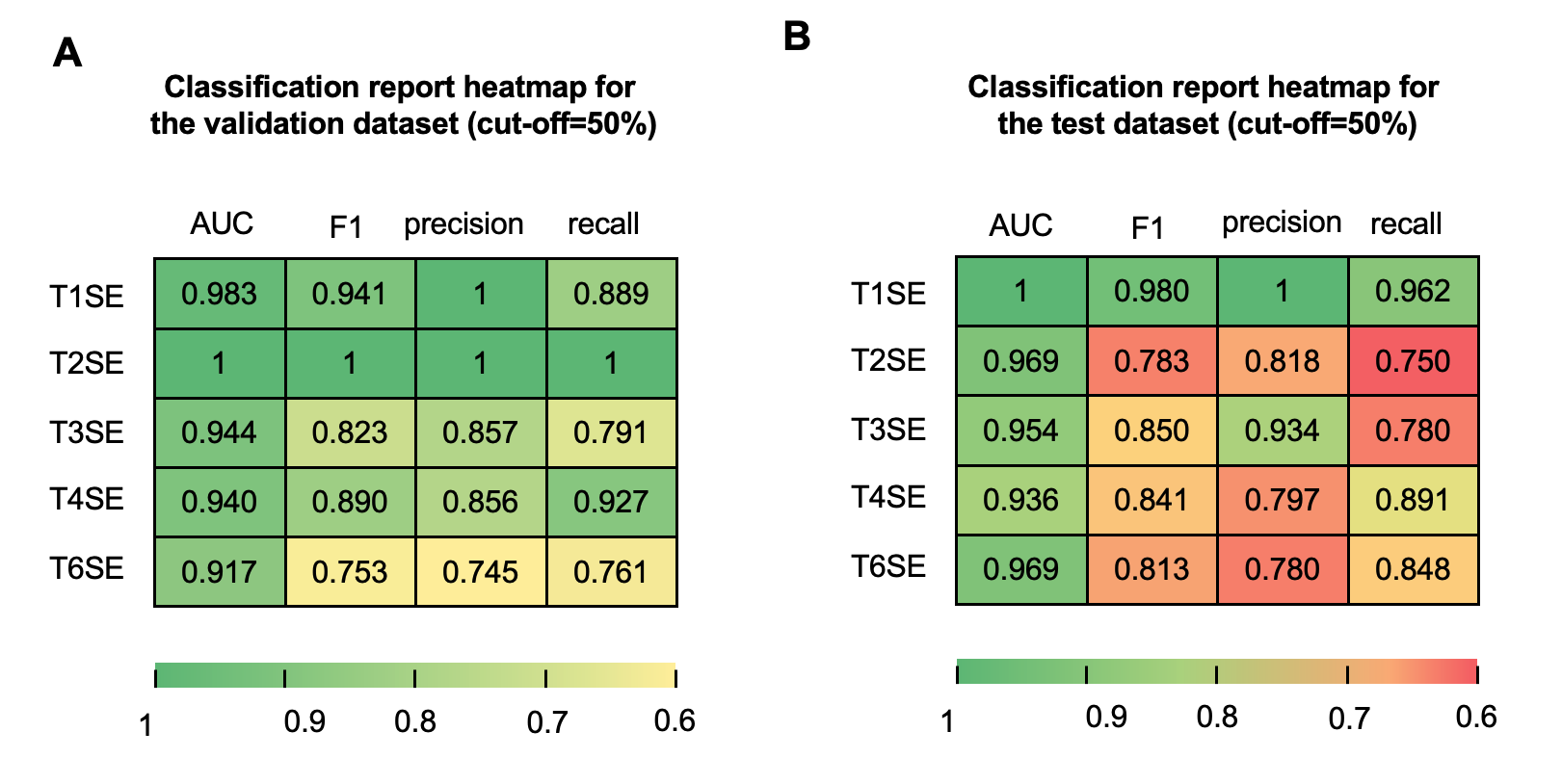

Supplement: S14 Fig — (TIF) [file pcbi.1013677.s015.tif]
